# Supplementary figures and images for: Mitochondrial Complex I and ROS control neuromuscular function through opposing pre- and postsynaptic mechanisms
Source: PLoS Biol. 2025 Sep 22;23(9):e3003388. doi: 10.1371/journal.pbio.3003388 (PMC12478897; doi:10.1371/journal.pbio.3003388)

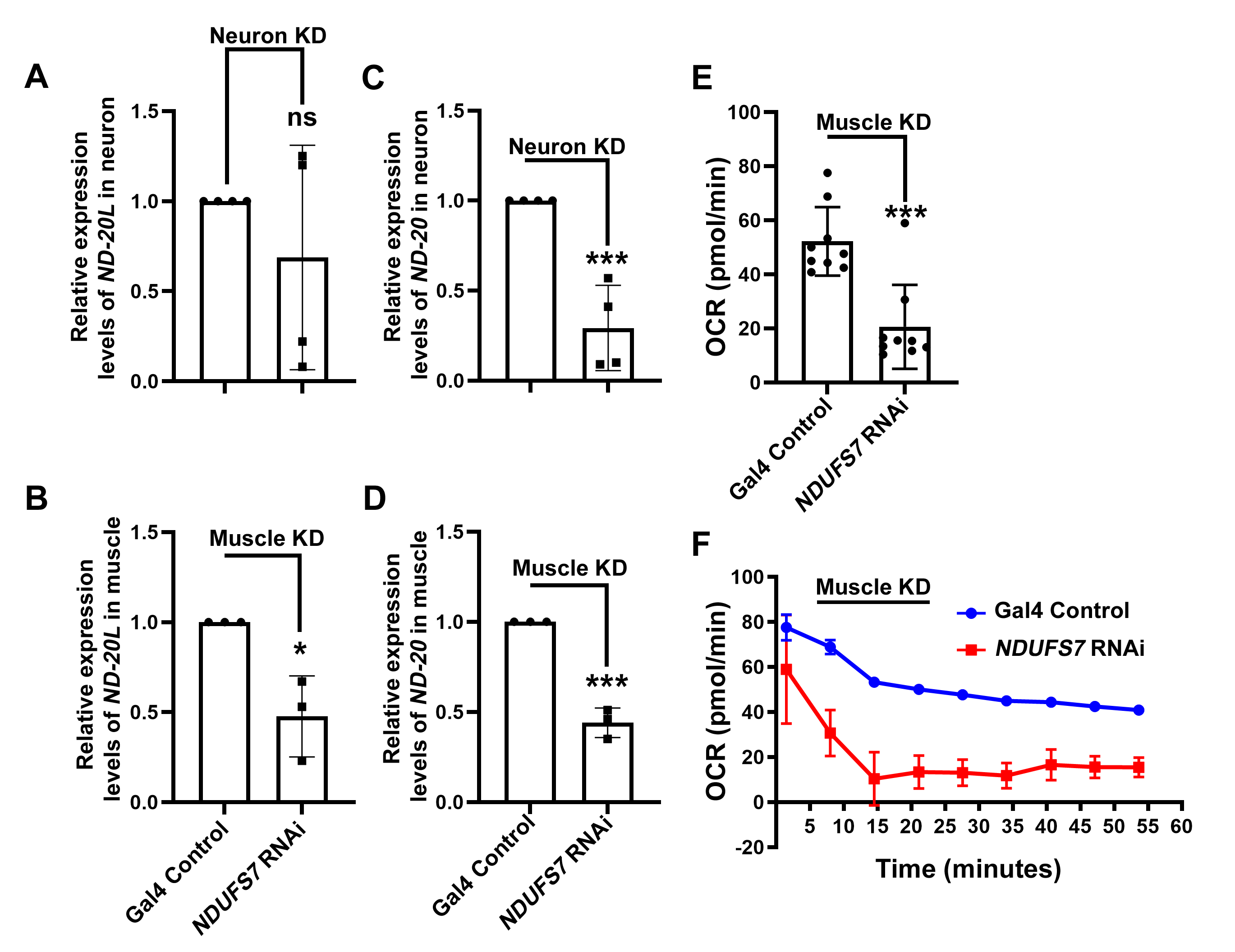

Supplement: S1 Fig — (A and B) Quantitative RT-PCR showing transcript levels of ND-20L in Gal4 controls and pan-neuronal and muscle Gal4-driven UAS-NDUFS7[RNAi]. Compared to pan-neuronal Gal4 control (elaV(C155)-Gal4/+), elaV(C155)-Gal4-driven UAS-NDUFS7[RNAi] (elaV(C155)-Gal4/+; NDUFS7[RNAi]/+) led to ~25% reduction in ND-20L transcript level in neurons. The muscle Gal4-driven UAS-NDUFS7[RNAi] showed ~50% reduction in ND-20L transcript levels (UAS-NDUFS7[RNAi]/+; BG57-Gal4/+) compared to Gal4 control (BG57-Gal4/+) in the muscle. Error bars represent mean ± standard deviation p = 0.354, *p = 0.015. Statistical analysis based on Student t test for pairwise comparisons. (C and D) Quantitative RT-PCR showing transcript levels of ND-20 in Gal4 controls and pan-neuronal and muscle Gal4-driven UAS-NDUFS7[RNAi]. Compared to pan-neuronal Gal4 control (elaV(C155)-Gal4/+), elaV(C155)-Gal4-driven UAS-NDUFS7[RNAi] (elaV(C155)-Gal4/+; UAS-NDUFS7[RNAi]/+) led to ~70% reduction in ND-20 transcript level in neurons. The muscle Gal4-driven UAS-NDUFS7[RNAi] showed ~60% reduction in ND-20 transcript levels (UAS-NDUFS7[RNAi]/+; BG57-Gal4/+) compared to Gal4 control (BG57-Gal4/+) in the muscle. Error bars represent mean ± standard deviation ***p = 0.001, ***p = 0.0003. Statistical analysis based on Student's t test for pairwise comparisons. (E) Histogram showing OCR in the mitochondria isolated from the thoracic region in muscle Gal4 control and muscle-depleted UAS-NDUFS7[RNAi] animals. The muscle depletion of NDUFS7 message resulted in a ~80% reduction in oxygen consumption compared to the Gal4 control. (F) OCR in muscle Gal4 control and muscle-depleted UAS-NDUFS7[RNAi] are plotted on a 60-min time scale. Raw data for this figure are available in the S2 Data Excel file, tab S1 Fig. (TIF) [file pbio.3003388.s004.tif]

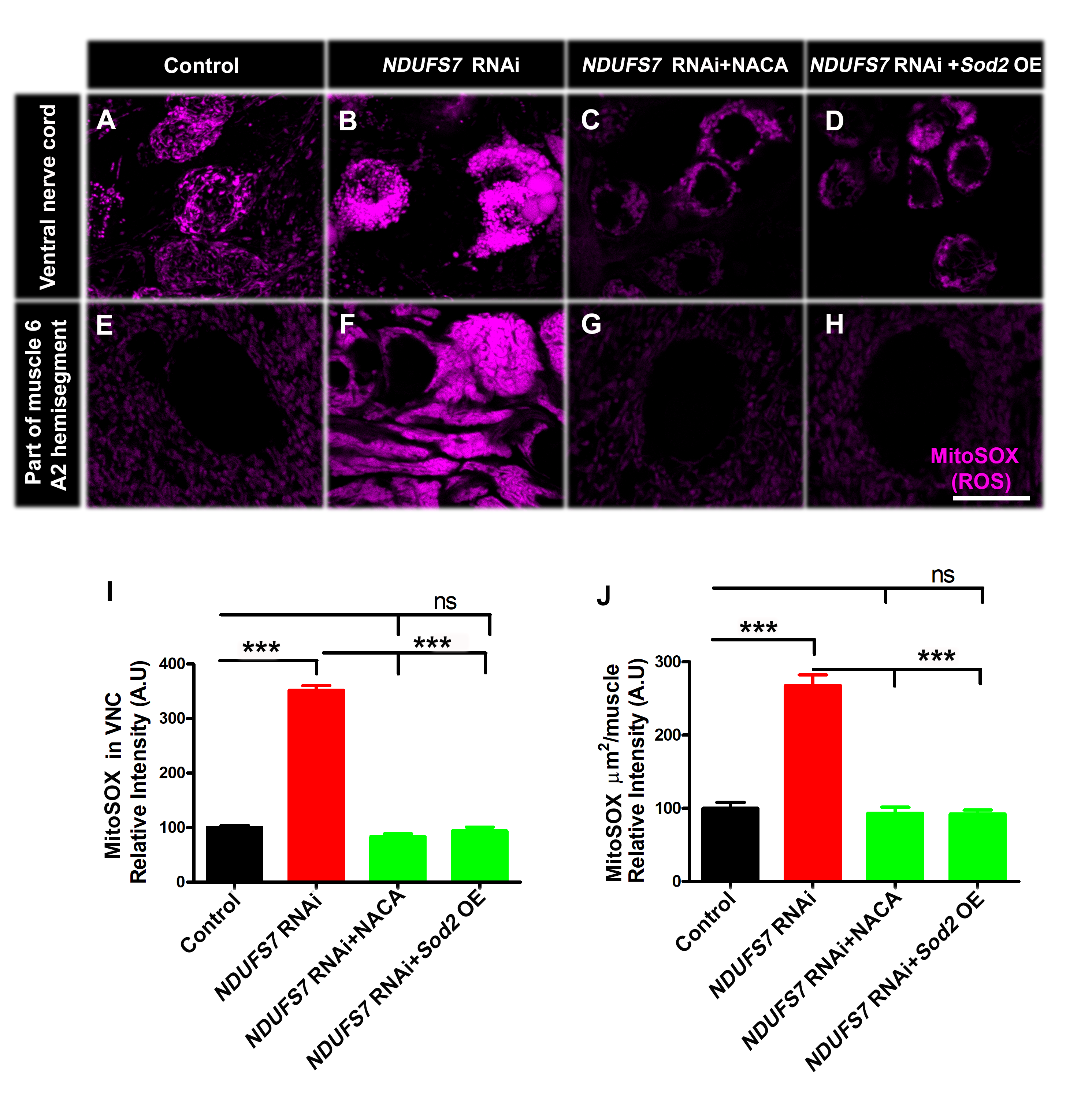

Supplement: S2 Fig — (A–D) Representative confocal images of the ventral nerve cord (VNC) at the third instar larval brain in (A) mito-GFP, D42-Gal4/+, (B) UAS-NDUFS7[RNAi]/+; mito-GFP, D42-Gal4/+, (C) UAS-NDUFS7[RNAi]/+; mito-GFP, D42-Gal4/+ with NACA, and (D) UAS-NDUFS7[RNAi]/UAS-Sod2; mito-GFP, D42-Gal4/+ labeled with superoxide indicator MitoSOX (magenta) in live animals. UAS-NDUFS7[RNAi]-depleted larvae supplemented with NACA or co-expressing Sod2 in neurons showed a significant correction in mitochondrial ROS levels compared to the NDUFS7[RNAi] knockdown tissues. (E–H) Confocal images of third instar larval body wall muscle 6 at A2 hemisegment in the indicated genotypes. Muscles depleted of MCI function by UAS-NDUFS7[RNAi] had ROS corrected back down to normal levels when the animals were supplemented with NACA or when there was co-expression of a UAS-Sod2 transgene. Scale bar: 5 μm. (I and J) Histogram showing the relative intensity of mitoSOX in VNC and body wall muscle in (E) UAS-mitoGFP/+; BG-57-Gal4/+, (F) UAS-NDUFS7[RNAi]/UAS-mitoGFP; BG57-Gal4/+, (G) UAS-NDUFS7[RNAi]/UAS-mitoGFP; BG-57-Gal4/+, and (H) UAS-NDUFS7[RNAi]/UAS-Sod2; BG57-Gal4/+ labeled with superoxide indicator MitoSOX (magenta) in live animals. ***p < 0.0001; ns, not significant. Statistical analysis based on one-way ANOVA followed by post-hoc Tukey’s multiple-comparison test. Error bars represent mean ± s.e.m. Raw data for this figure are available in the S2 Data Excel file, tab S2 Fig. (TIF) [file pbio.3003388.s005.tif]

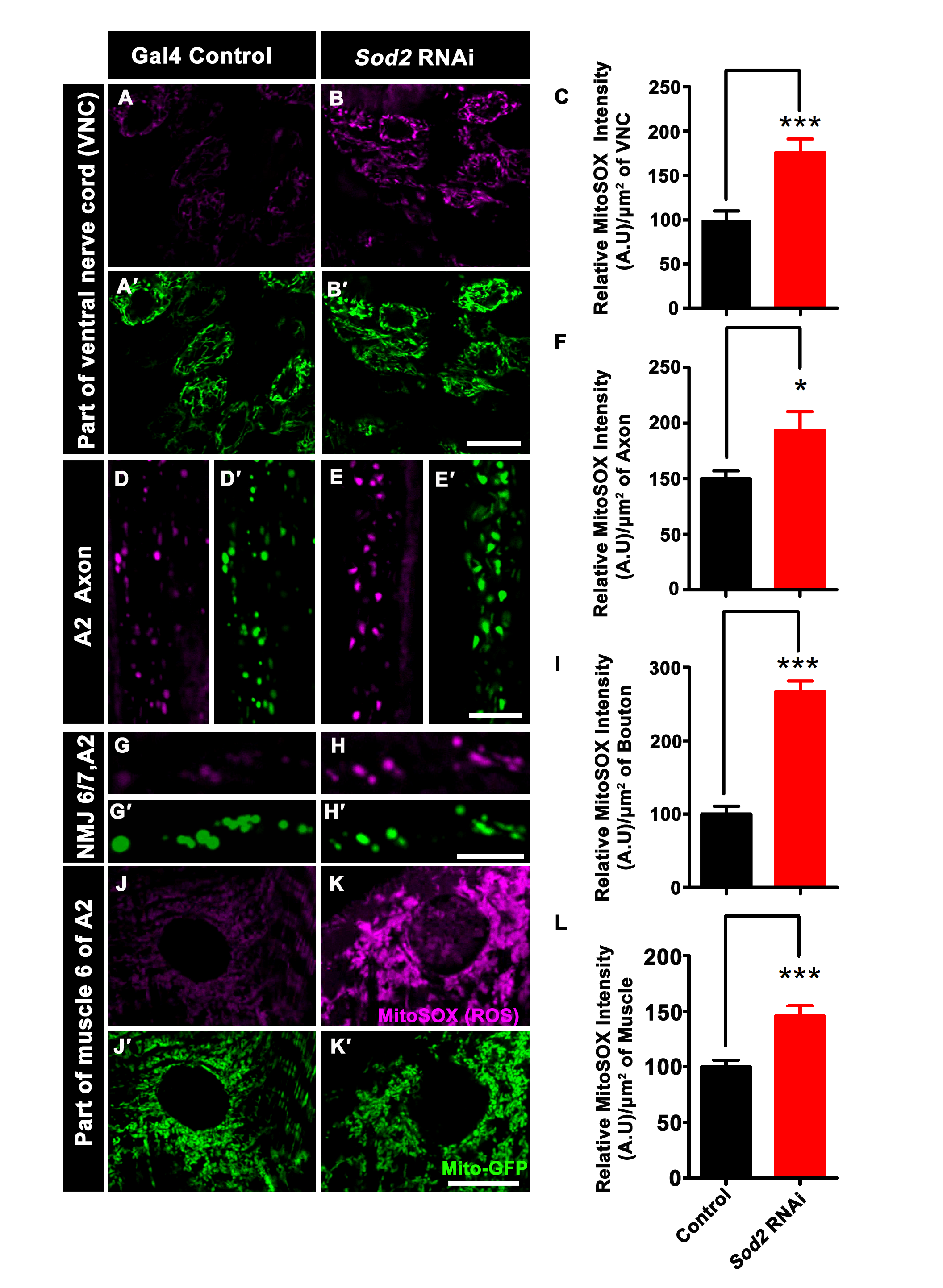

Supplement: S3 Fig — (A, -A′ and B, B′) Representative confocal images of the ventral nerve cord (VNC) at the third instar larval brain in the genotypes (A, A′) UAS-mito-GFP, D42-Gal4/+ and (B, B′) UAS-mito-GFP, D42-Gal4/Sod2[RNAi] labeled with superoxide indicator MitoSOX (magenta) and mito-GFP (green) in live tissue. (C) Histogram showing the relative intensity of MitoSOX in the VNC of indicated genotypes. ***p = 0.0003 (VNC: control versus Sod2[RNAi]). (D, D′ and E, E′) Representative confocal images of the axon at the third instar larval fillet in (D, D′) UAS-mito-GFP, D42-Gal4/+ and (E, E′) UAS-mito-GFP, D42-Gal4/UAS-Sod2[RNAi] labeled with superoxide indicator MitoSOX (magenta) and mitoGFP (green) in live tissue. (F) Histogram showing the relative intensity of MitoSOX in axons in the indicated genotypes. *p = 0.020 (Axon: control versus Sod2 RNAi). (G, G′ and H, H′) Representative confocal images of a third instar bouton of synapse 6/7 in the A2 hemisegment in (G, G′) UAS-mito-GFP, D42-Gal4/+, and (H, H′) UAS-mito-GFP, D42-Gal4/Sod2[RNAi] labeled with superoxide indicator MitoSOX (magenta) and mitoGFP (green) in live tissue. (I) Histogram showing the relative intensity of MitoSOX at boutons in the indicated genotypes. ***p < 0.0001 (boutons: control versus Sod2 RNAi). (J, J′ and K, K′) Representative confocal images of the third instar 6/7 muscle of A2 hemi segment in (J-J′) UAS-mito-GFP/+; BG57-Gal4/+, and (K, K′) UAS-mito-GFP/+; BG57-Gal4/Sod2[RNAi] labeled with superoxide indicator MitoSOX (magenta) and mitoGFP (green) in live animals. (L) Histogram showing the relative intensity of mitoSOX in 6/7 muscle in the indicated genotypes. ***p = 0.0001 (6/7 muscles: control versus Sod2 [RNAi]). The depletion of Sod2 by RNAi results in the abnormal accumulation of reactive oxygen species (ROS) in neurons and muscles. Scale bar: 10 μm (A, A′–E, E′) and 5 μm (G, G′ and K, K′). Statistical analysis based on Student’s t test for pairwise comparison. Error bars represent mean ± s.e.m. Raw d [file pbio.3003388.s006.tif]

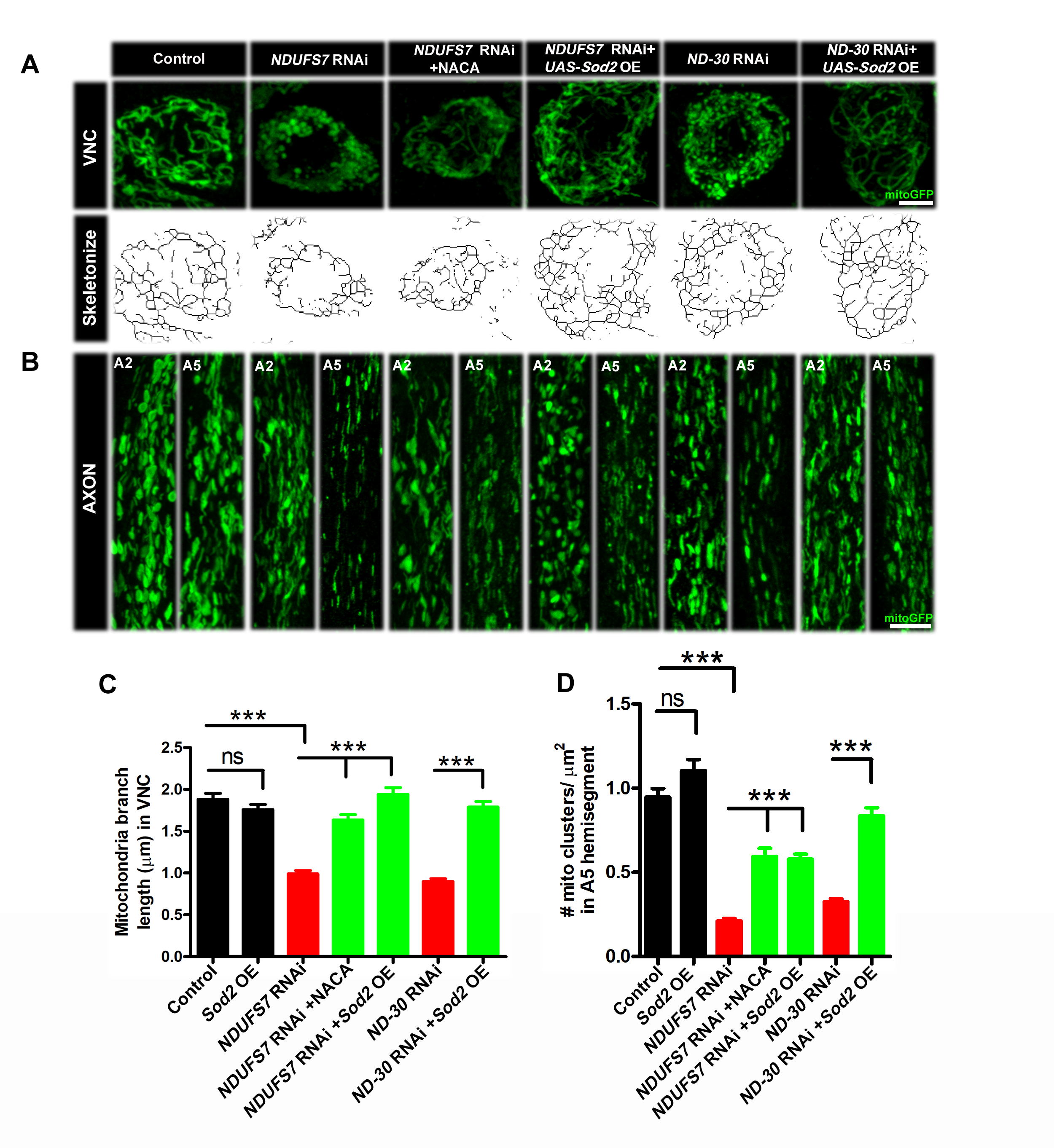

Supplement: S4 Fig — UAS-NDUFS7[RNAi], UAS-NDUFS7[RNAi] co-expressing UAS-Sod2, ND-30[RNAi], and ND-30[RNAi] co-expressing UAS-Sod2 and controls were crossed to a motor neuron driver line (D42-Gal4, UAS-mitoGFP) to label neuronal mitochondria. (A) Ventral nerve cord (VNC): UAS-mitoGFP exhibits normal mitochondrial organization, UAS-NDUFS7[RNAi] and ND-30 RNAi exhibit clustered mitochondria. However, when UAS-NDUFS7[RNAi] animals were raised in a media containing NACA or when there was co-expression of UAS-Sod2 in UAS-NDUFS7[RNAi]- and ND-30[RNAi]-depleted animals, there was a restoration of normal mitochondrial organization. The respective fluorescent images were skeletonized to measure mitochondrial branch length (organization). (B) Comparison of a proximal axonal segment in A2 and a distal segment in A5. Distal segments of A5 axons in UAS-NDUFS7[RNAi] contain fewer mitochondria than proximal segments. Scale bar: 5 μm. Mitochondrial distribution was significantly suppressed back to control values when UAS-NDUFS7[RNAi] was raised in media containing NACA or genetically over-expressing UAS-Sod2 in the UAS-NDUFS7[RNAi] and ND-30[RNAi] backgrounds in neurons. (C and D) Histogram showing mitochondrial branch length (μm) and number in the indicated genotypes. ***p < 0.0001, ns, not significant. Statistical analysis based on one-way ANOVA followed by post-hoc Tukey’s multiple-comparison test. Error bars represent mean ± s.e.m. Raw data for this figure are available in the S2 Data Excel file, tab S4 Fig. (TIF) [file pbio.3003388.s007.tif]

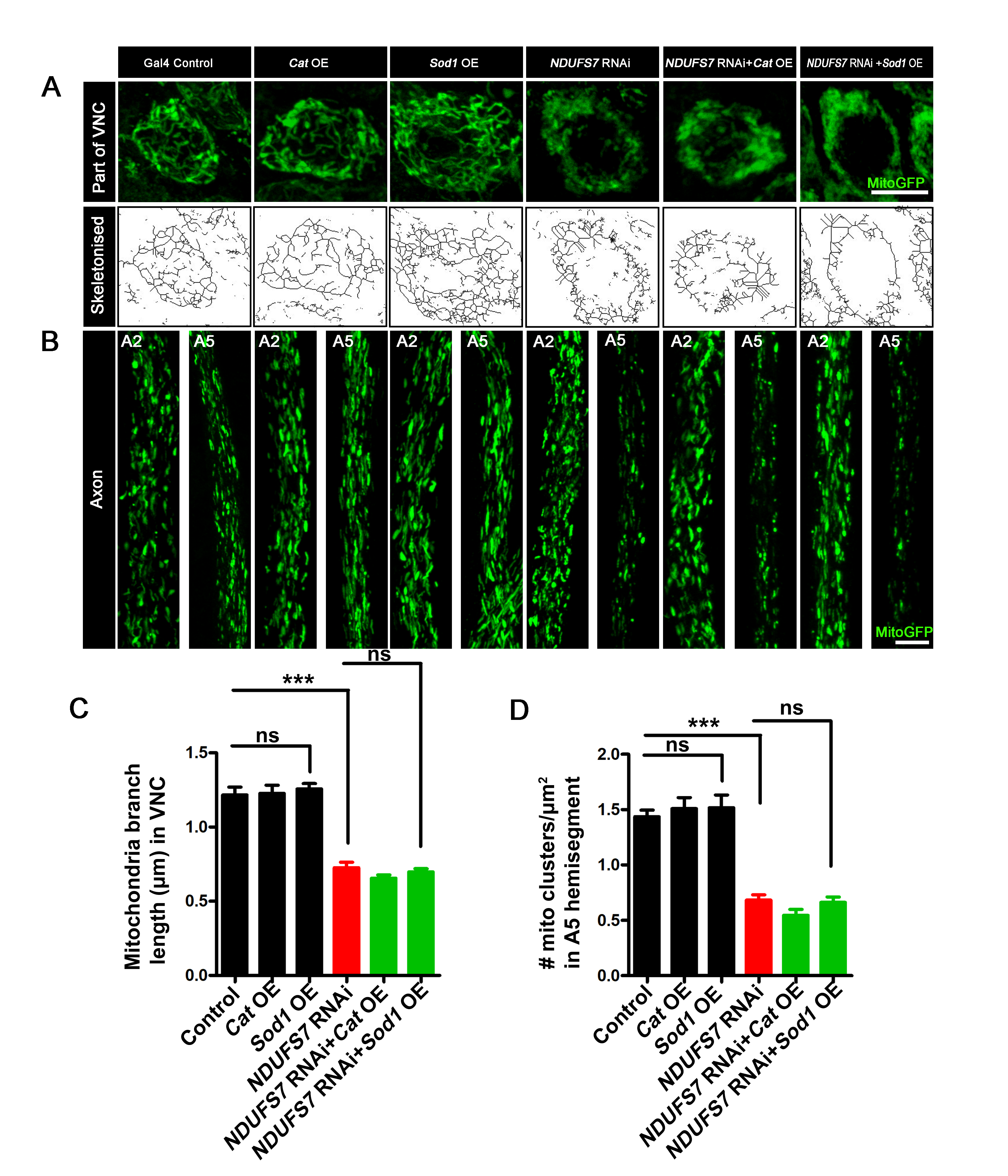

Supplement: S5 Fig — Mitochondrial morphology and distribution are affected in the ventral nerve cord and distal axons. Drosophila stocks of UAS-NDUFS7[RNAi], UAS-NDUFS7[RNAi] co-expressing UAS-Catalase or UAS-Sod1, as well as controls,were crossed to a motor neuron driver (D42-Gal4, UAS-mitoGFP) to label neuronal mitochondria. (A) Ventral nerve cord (VNC): UAS-mitoGFP, UAS-Cat, and UAS-Sod1 exhibit normal mitochondrial organization. By contrast, NDUFS7[RNAi] exhibits clustered mitochondria. Co-expression of UAS-Cat or UAS-Sod1 in the NDUFS7[RNAi] background does not restore normal mitochondrial organization. The respective fluorescent images were skeletonized to measure mitochondrial branch length (organization). Scale bar: 10 μm. (B) Comparison of a proximal axonal segment in A2 and a distal segment in A5. Distal segments of A5 axons in NDUFS7[RNAi] contain fewer mitochondria than proximal segments. Scale bar: 5 μm. Mitochondrial distribution was not restored to control values when NDUFS7[RNAi] was coexpressed with UAS-Cat or UAS-Sod1 in neurons. (C and D) Histogram showing mitochondrial branch length (μm) and number in the indicated genotypes. ***p < 0.0001, ns, not significant. Statistical analysis based on one-way ANOVA followed by post-hoc Tukey’s multiple-comparison test. Error bars represent mean ± s.e.m. (TIF) [file pbio.3003388.s008.tif]

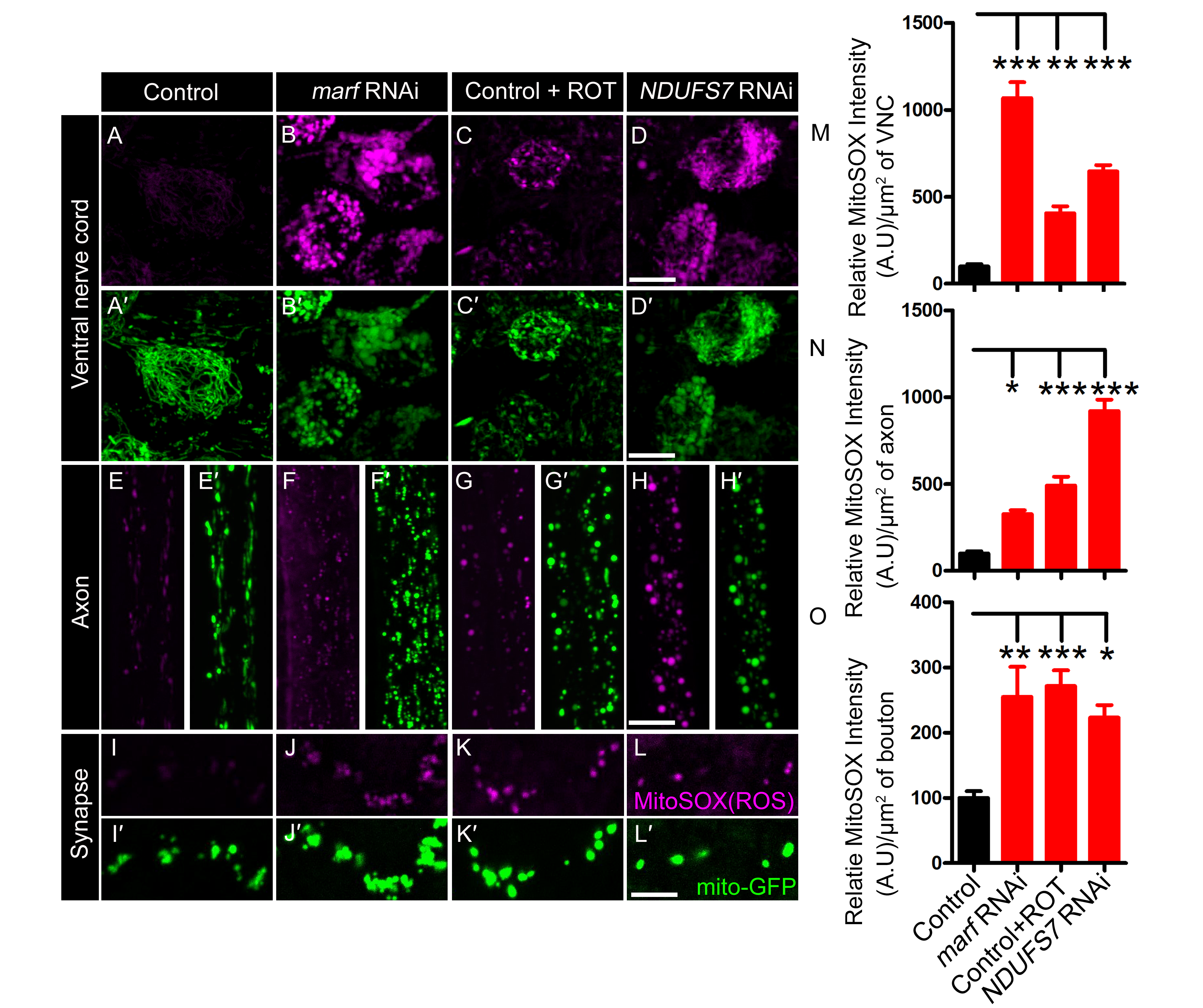

Supplement: S6 Fig — (A, A′–D, D′) Representative confocal images of the ventral nerve cord (VNC) at the third instar larval brain in (A, A′) mito-GFP, D42-Gal4/+, (B, B′) mito-GFP, D42-Gal4/marf[RNAi], (C, C′) mito-GFP, D42-Gal4/+ with 25 μM rotenone (ROT), and (D, D′) UAS-NDUFS7[RNAi]/+; mito-GFP, D42-Gal4/+ labeled with superoxide indicator MitoSOX (magenta) and mitoGFP (green) in live animals. (E, E′–H, H′) Representative confocal images of the axon at the third instar larval fillet in (E, E′) mito-GFP, D42-Gal4/+, (F, F′) mito-GFP, D42-Gal4/marf[RNAi], (G, G′) mito-GFP, D42-Gal4/+ with 25 μM ROT and (H, H′) UAS-NDUFS7[RNAi]/+; mito-GFP, D42-Gal4/+ labeled with superoxide indicator MitoSOX (magenta) and mitoGFP (green) in live animals. (I, I′–L, L′). Representative confocal images of the third instar bouton of A2 hemisegment in (II’) mito-GFP, D42-Gal4/+, (J, J′) mito-GFP, D42-Gal4/marf[RNAi], (K, K′) mito-GFP, D42-Gal4/+ with 25 μM ROT and (L-L’) UAS-NDUFS7[RNAi]/+; mito-GFP, D42-Gal4/+ labeled with superoxide indicator MitoSOX (magenta) and mitoGFP (green) in live animals. The depletion of marf by RNAi or blocking MCI activity induces abnormal accumulation of ROS in neurons. Scale bar: 10 μm. (M-O) Histogram showing the relative intensity of MitoSOX in VNC, axon and boutons in the indicated genotypes ***p < 0.0001; **p < 0.001; **p = 0.002 (Bouton: control versus marf[RNAi]), *p < 0.05. Statistical analysis based on one-way ANOVA followed by post-hoc Tukey’s multiple-comparison test. Error bars represent mean ± s.e.m. Raw data for this figure are available in the S2 Data Excel file, tab S6 Fig. (TIF) [file pbio.3003388.s009.tif]

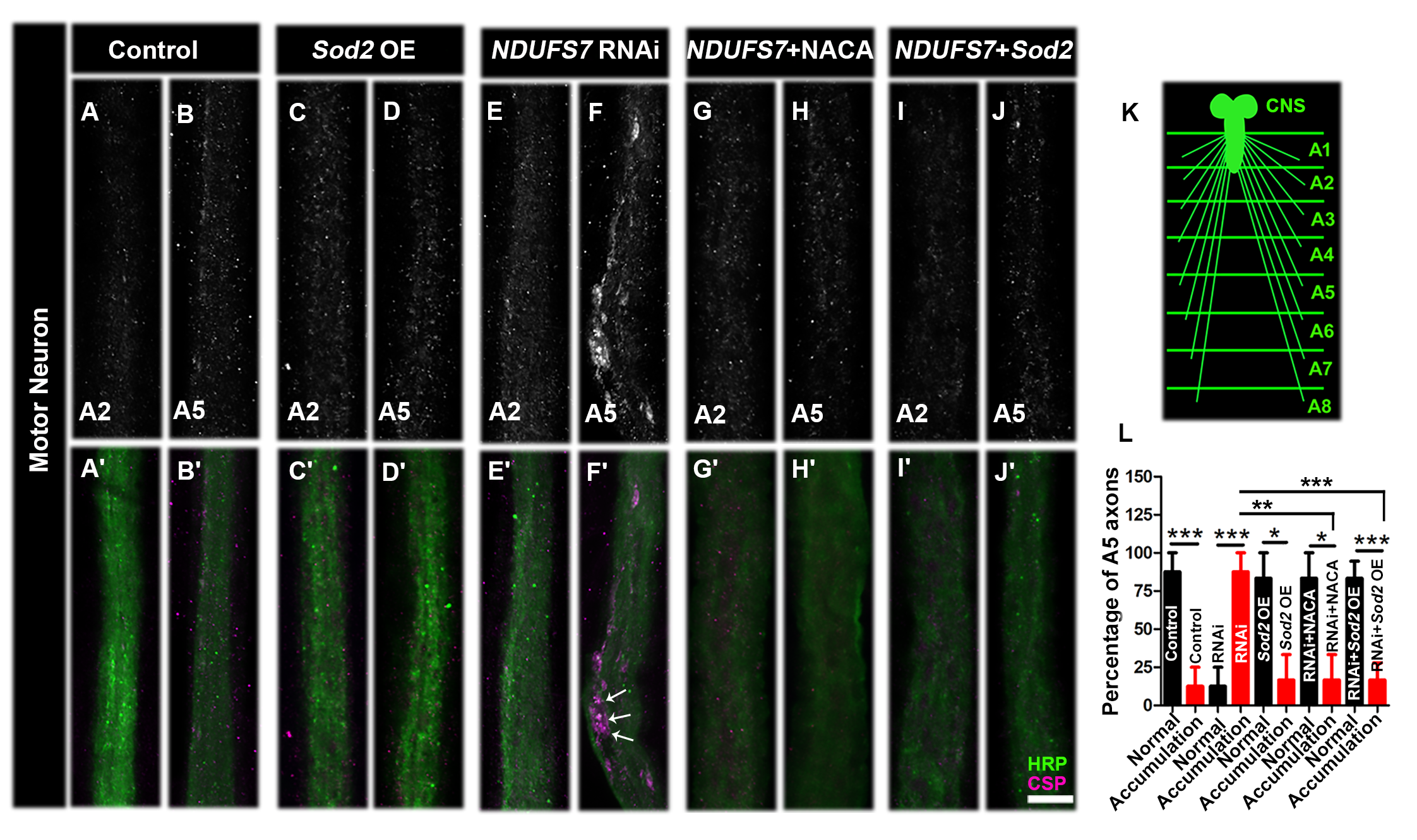

Supplement: S7 Fig — Representative confocal images of the proximal (A2) and distal (A5) axons of larvae in (A, A′ and B, B′) Gal4 control, (C, C′ and D, D′) UAS-Sod2/+; D42-Gal4/+, (E, E′ and F, F′) UAS-NDUFS7[RNAi]/+; D42-Gal4/+, (G, G′ and H, H′) UAS-NDUFS7[RNAi]/+; D42-Gal4/+ with NACA, and (I, I′ and J, J′) UAS-NDUFS7[RNAi]/UAS-Sod2; D42-Gal4/+. Axons were double immunolabeled with CSP (magenta) and HRP (green) antibodies. Motor neuron-depleted UAS-NDUFS7[RNAi] larval axons showed abnormal accumulation of CSP at a more distal hemisegment (A5) compared to Gal4 controls. Scale bar: 10 μm. CSP aggregates were cleared when UAS-NDUFS7[RNAi] animals were raised in media containing NACA or genetically expressing UAS-Sod2 in neurons. (K) Schematic illustration showing VNC, axons and body wall muscle in a third instar larvae (L) Histogram showing the percentage of axons with abnormal accumulation in the indicated genotypes. ***p = 0.0008 (control: normal versus accumulation), *p = 0.0008 (UAS-NDUFS7[RNAi]: normal versus accumulation), *p = 0.0179 (Sod2 OE: normal versus accumulation), *p = 0.0179 (UAS-NDUFS7[RNAi] with NACA: normal versus accumulation), *p = 0.0004 (UAS-NDUFS7[RNAi] with Sod2 OE: normal versus accumulation), **p = 0.0046 (UAS-NDUFS7[RNAi] versus UAS-NDUFS7[RNAi] with NACA: accumulation) and ***p = 0.0006 (UAS-NDUFS7[RNAi] versus UAS-NDUFS7[RNAi] with Sod2 OE: accumulation). Statistical analysis was based on Fisher’s exact test to differentiate two distinct phenotypes in the same sample. Error bars represent mean ± s.e.m. Raw data for this figure are available in the S2 Data Excel file, tab S7 Fig. (TIF) [file pbio.3003388.s010.tif]

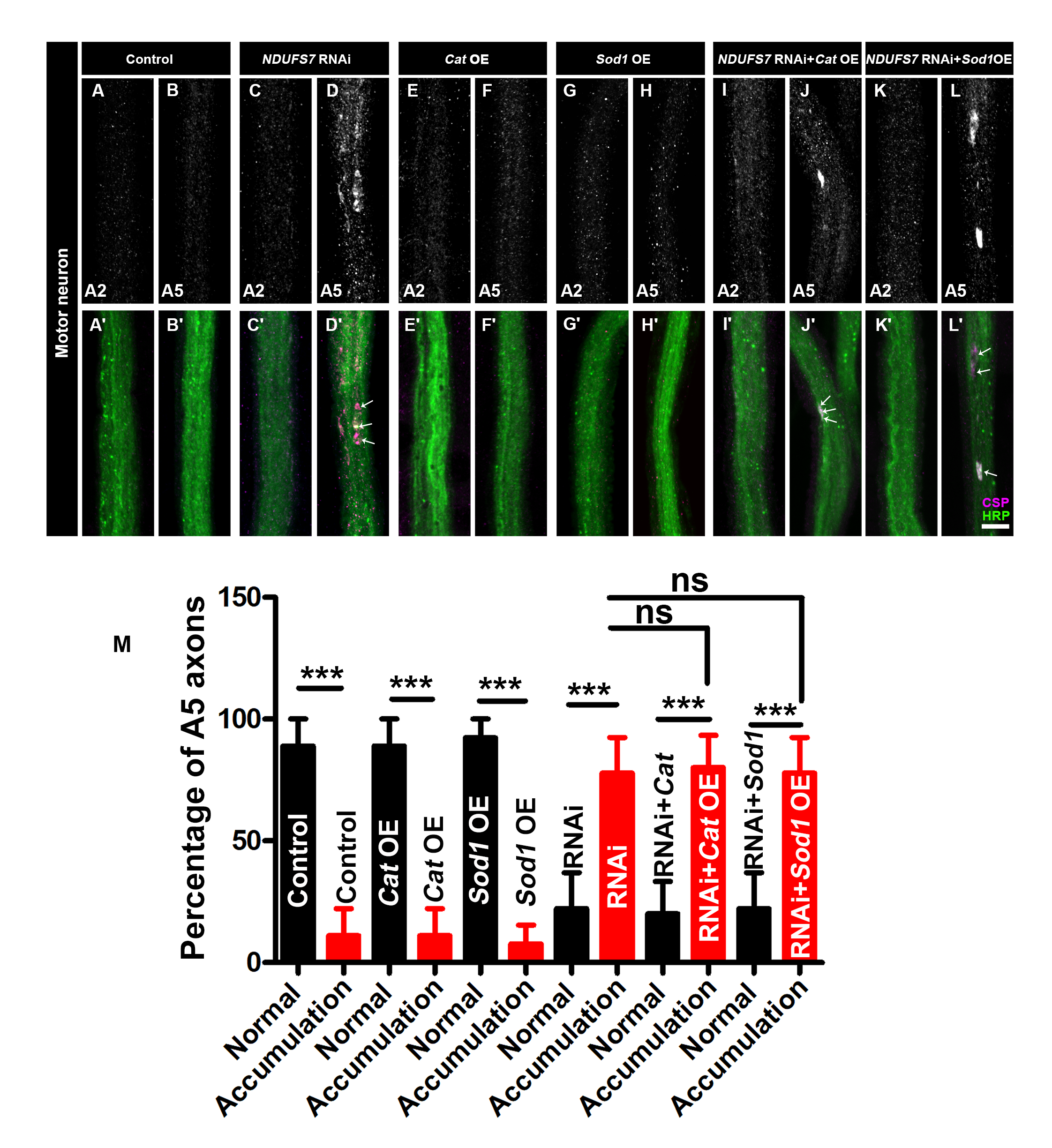

Supplement: S8 Fig — Representative confocal images of the proximal (A2) and distal (A5) axons of larvae in (A, A′, B, B′) Gal4 control: UAS-mitoGFP, D42-Gal4, (C, C′, D, D′) NDUFS7[RNAi]/+; UAS-mitoGFP, D42-Gal4/+, (E, E′, F, F′) UAS-Cat/+; UAS-mitoGFP, D42-Gal4/+, (G, G′, H, H′) UAS-Sod1/+; UAS-mitoGFP, D42-Gal4/+, (I, I′, J, J′) NDUFS7[RNAi]/UAS-Cat; UAS-mitoGFP, D42-Gal4/+, and (J, J′, K, K′) NDUFS7[RNAi]/UAS-Sod1; UAS-mitoGFP, D42-Gal4/+. Axons were double immunolabeled with CSP (magenta) and HRP (green) antibodies. Motor neuron-depleted NDUFS7[RNAi] larval axons showed abnormal accumulation of CSP in axons at the more distal hemisegment (A5) compared to Gal4 controls. Scale bar: 10 μm. CSP aggregates were not cleared when NDUFS7[RNAi] animals were concurrently expressing UAS-Cat or UAS-Sod1 in neurons. (M) Histogram showing the percentage of axons with abnormal accumulation in the indicated genotypes. ***p < 0.0001 (control: normal vs. accumulation), ***p < 0.0001 (NDUFS7[RNAi]: normal vs. accumulation), ***p < 0.0001 (Cat OE: normal vs. accumulation), ***p < 0.0001 (Sod1 OE: normal vs. accumulation), ***p < 0.0001 (NDUFS7[RNAi]+Cat OE: normal vs. accumulation), ***p < 0.0001 (NDUFS7[RNAi]+Sod1 OE: normal vs. accumulation), p = 0.911 (NDUFS7[RNAi] vs. NDUFS7[RNAi]+Cat OE: accumulation) and p = 1.00 (NDUFS7[RNAi] vs. NDUFS7[RNAi]+Sod1 OE: accumulation). Statistical analysis was based on Fisher’s exact test to differentiate two distinct phenotypes in the same sample. Error bars represent mean ± s.e.m. Raw data for this figure are available in the S2 Data Excel file, tab S8 Fig. (TIF) [file pbio.3003388.s011.tif]

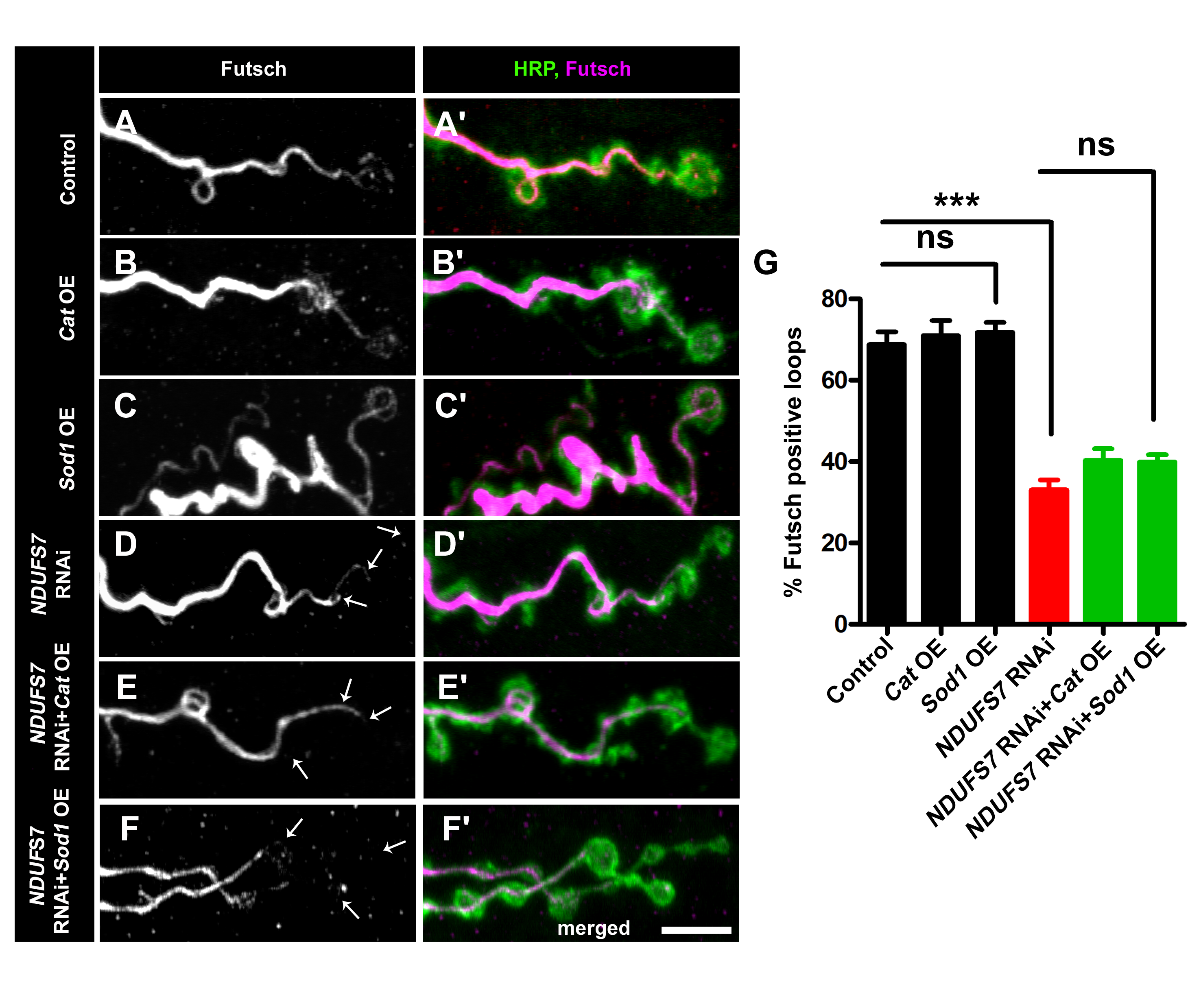

Supplement: S9 Fig — Representative confocal images of NMJ synapses at muscle 6/7 of (A, A′) UAS-mitoGFP, D42-Gal4 control, (B, B′) UAS-Cat overexpression (UAS-Cat/+; UAS-mitoGFP, D42-Gal4/+), (C, C′) UAS-Sod1 overexpression (UAS-Sod1/+; UAS-mitoGFP, D42-Gal4/+), (D, D′) UAS-mitoGFP, D42-Gal4-driven NDUFS7[RNAi] (UAS-NDUFS7[RNAi]/+; UAS-mitoGFP, D42-Gal4/+), (E, E′) NDUFS7 knockdown with UAS-Cat (UAS-NDUFS7[RNAi]/UAS-Cat; UAS-mitoGFP, D42-Gal4/+) and (F, F′) NDUFS7 knockdown with UAS-Sod1 (UAS-NDUFS7[RNAi]/UAS-Sod1; UAS-mitoGFP, D42-Gal4/+). Each condition was double immunolabeled with 22C10 (anti-Futsch, magenta) and anti-HRP (green) antibodies. The motor neuron-depleted NDUFS7[RNAi] larvae showed a decrease in the number of Futsch-positive loops as compared to the Gal4 control. Neither UAS-Cat nor UAS-Sod1 restored Futsch-positive loops in the UAS-NDUFS7[RNAi] background. Scale bar: 5 μm. (G) Histograms showing the percentage of Futsch-positive loops in the indicated genotypes. p = 0.659 (Control versus UAS-Cat), p = 0.505 (Control versus UAS-Sod1), p < 0.0001 (Control versus UAS-NDUFS7[RNAi]), p = 0.071 (UAS-NDUFS7[RNAi] versus UAS-NDUFS7[RNAi] + UAS-Cat) and p = 0.039 (UAS-NDUFS7[RNAi] versus UAS-NDUFS7[RNAi] + UAS-Sod1). Statistical analysis based on one-way ANOVA followed by post-hoc Tukey’s multiple-comparison test. Error bars represent mean ± s.e.m. Raw data for this figure are available in the S2 Data Excel file, tab S9 Fig. (TIF) [file pbio.3003388.s012.tif]

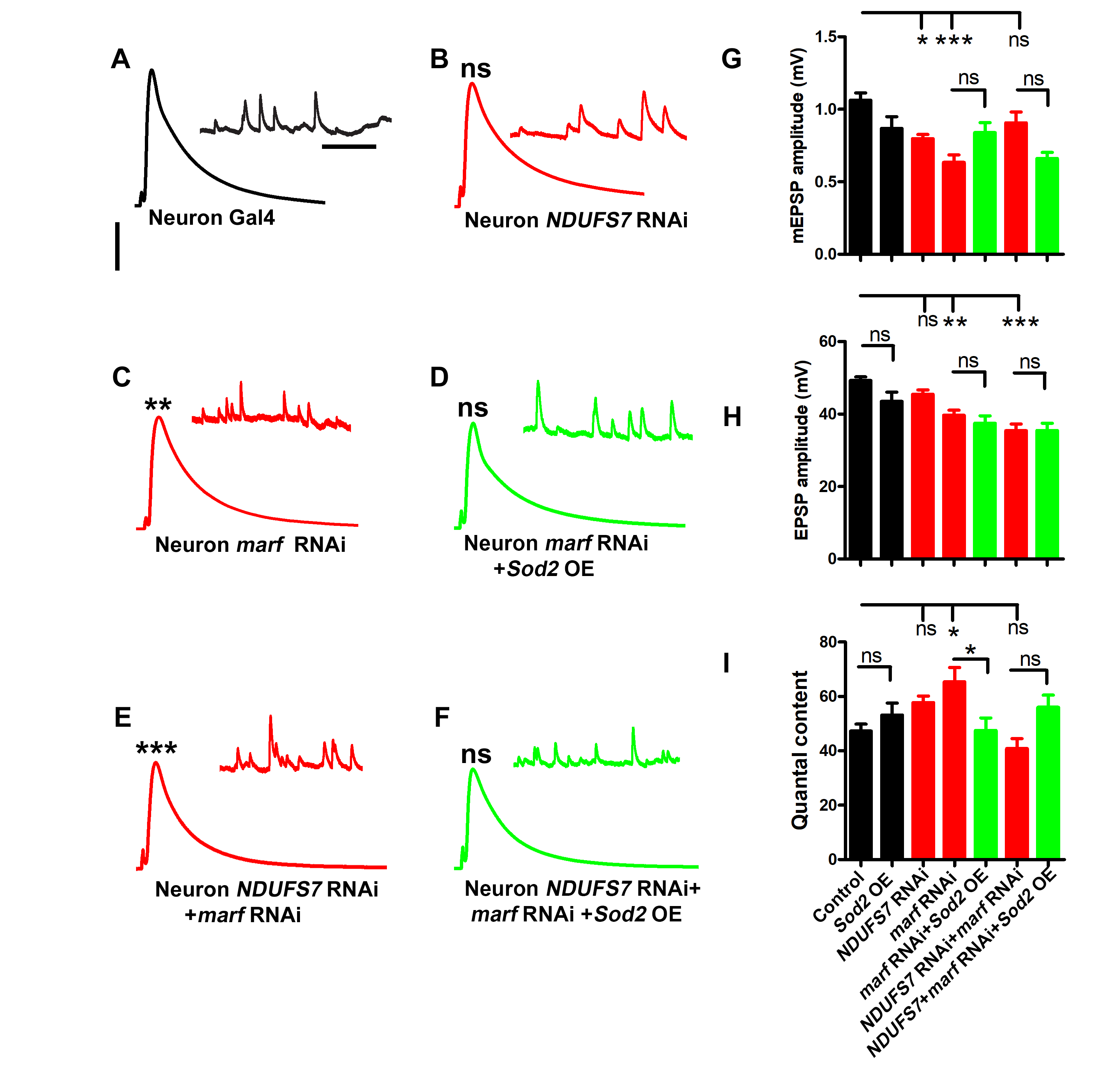

Supplement: S10 Fig — (A–F) Representative electrophysiological traces of mEPSPs and EPSPs in (A) motor-neuron Gal4 control D42-Gal4/+, (B) UAS-NDUFS7[RNAi]/+; D42-Gal4/+, (C) UAS-marf[RNAi]/D42-Gal4, (D) UAS-Sod2 OE/+; UAS-marf[RNAi]/D42-Gal4, (E) UAS-NDUFS7[RNAi]/+; UAS-marf[RNAi]/D42-Gal4, and (F) UAS-NDUFS7[RNAi]/UAS-Sod2; UAS-marf[RNAi]/D42-Gal4. Scale bars for EPSPs (mEPSP) are x = 50 ms (1,000 ms) and y = 10 mV (1 mV). Note that mEPSP and EPSP amplitudes were reduced in motor neuron Gal4-driven UAS-marf[RNAi] and UAS-marf[RNAi] + UAS-NDUFS7[RNAi] animals. (G–I) Histograms showing average mEPSPs, EPSPs, and quantal content in the indicated genotypes. A minimum of 8 NMJ recordings of each genotype were used for quantification. *p < 0.05 (mEPSP amplitude: control vs. UAS-NDUFS7[RNAi]), *p = 0.007 (QC: control vs. UAS-marf[RNAi]),*p = 0.026 (QC: UAS-marf[RNAi] vs. UAS-marf[RNAi] + Sod2 OE), **p < 0.001, ***p < 0.0001, ns, not significant. Statistical analysis based on one-way ANOVA followed by post-hoc Tukey’s multiple-comparison test. Error bars represent mean ± s.e.m. Raw data for this figure are available in the S2 Data Excel file, tab S10 Fig. (TIF) [file pbio.3003388.s013.tif]

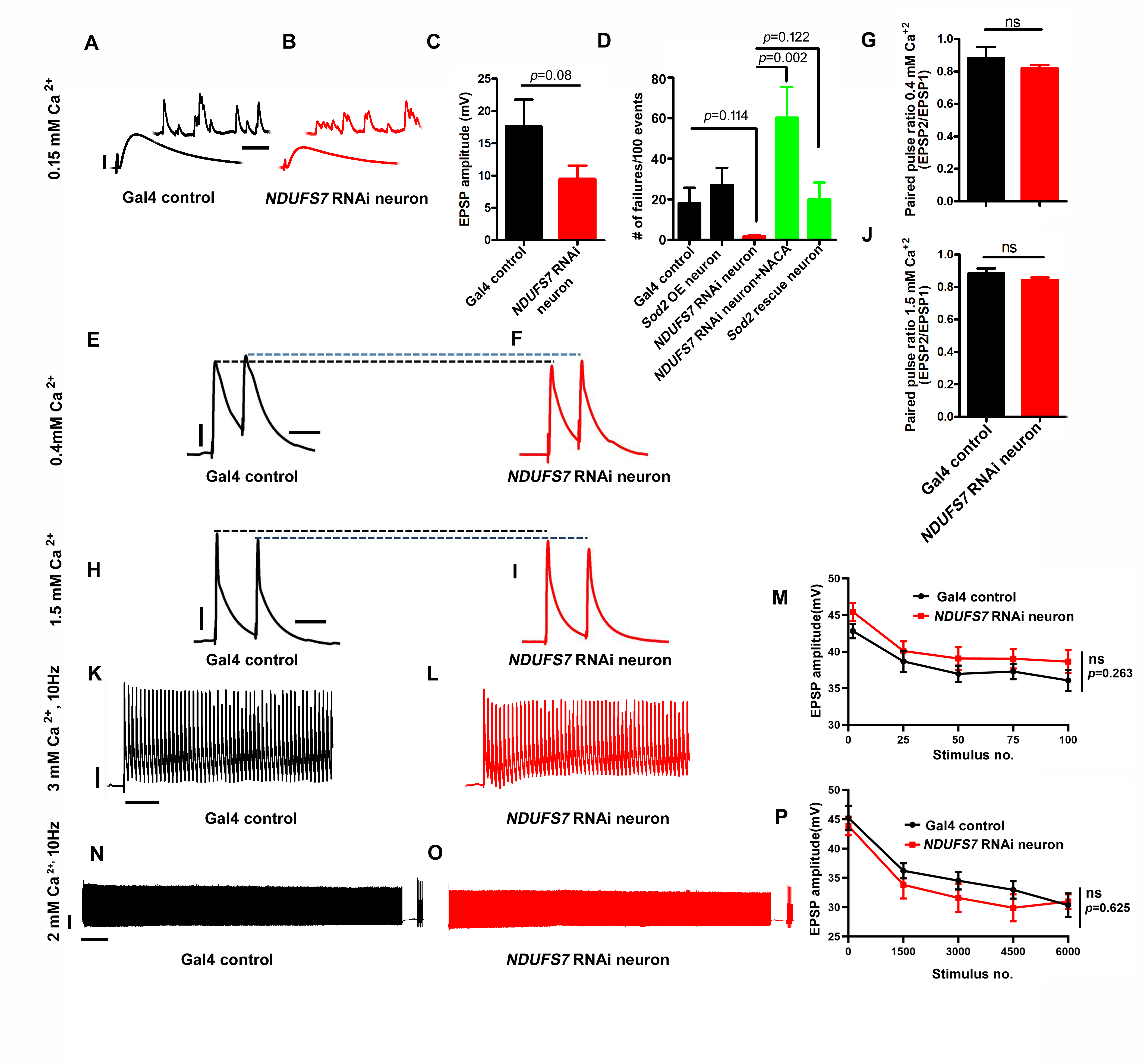

Supplement: S11 Fig — (A and B) Representative traces of EPSPs and mEPSPs in (A) motor neuron-Gal4 control (D42-Gal4/+) and (B) motor neuron-Gal4 driven UAS-NDUFS7[RNAi] (UAS-NDUFS7[RNAi]/+; D42-Gal4/+) at 0.15 mM extracellular Ca2+ concentration. Scale bars for EPSPs (mEPSP) are x = 50 ms (1,000 ms) and y = 10 mv (1 mV). (C) Quantification of EPSPs in the indicated genotypes. A very low extracellular calcium concentration mildly affects EPSPs in NDUFS7[RNAi] compared to control larvae. (D) Failure analysis at 0.1 mM Ca2+ for the following conditions: Gal4 control (D42-Gal4/+), Sod2 overexpression (UAS-Sod2/+; D42-Gal4/+), D42-Gal4-driven UAS-NDUFS7[RNAi] (UAS-NDUFS7[RNAi]/+; D42-Gal4/+), UAS-NDUFS7[RNAi]/+; D42-Gal4/+ with NACA, and UAS-NDUFS7[RNAi]/UAS-Sod2; D42-Gal4/+. The number of failures was counted per hundred trials in each genotype. There is a marked decrease in failure rate in motor neurons driving UAS-NDUFS7[RNAi]. The synaptic failure rates were restored back up to baseline levels when UAS-NDUFS7[RNAi]-depleted flies were reared in the media containing NACA or genetic expression of Sod2 in motor neurons. (E and F) Representative paired-pulse EPSP traces at 0.4 mM extracellular Ca2+ in the indicated genotypes. Scale bars for EPSPs are x = 50 ms and y = 10 mV. No change in paired-pulse ratio was observed in motor neuron-depleted UAS-NDUFS7[RNAi] animals at 0.4 mM Ca2+. (G) Quantification of paired-pulse ratio (EPSP2/EPSP1) in motor neuron-Gal4 control (D42-Gal4/+) and (B) motor neuron-Gal4 driven UAS-NDUFS7[RNAi] (UAS-NDUFS7[RNAi]/+; D42-Gal4/+) larvae. (H and I) Representative paired-pulse EPSP traces at 1.5 mM extracellular Ca2+ in the indicated genotypes. Scale bars for EPSPs are x = 50 ms and y = 10 mV. No change in paired-pulse ratio was observed in motor neuron-depleted UASNDUFS7[RNAi] animals, even at higher calcium concentrations. (J) Quantification of paired ratio (EPSP2/EPSP1) in motor neuron-Gal4 control (D42-Gal4/+) and (B) motor neuron-Gal4 driven UAS-NDUFS7[RNAi] [file pbio.3003388.s014.tif]

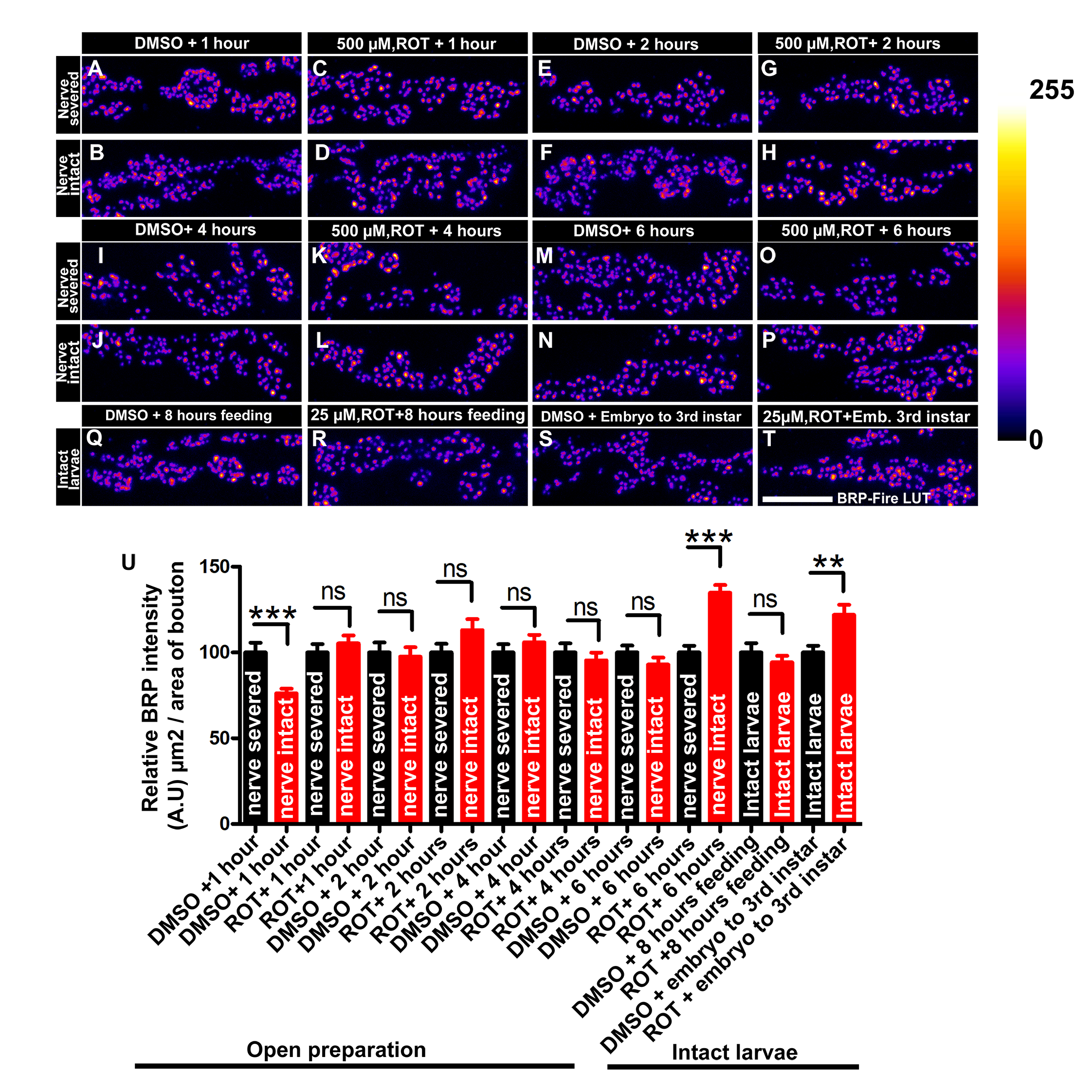

Supplement: S12 Fig — (A) Representative images of the A2 hemisegment of muscle 6/7 NMJs in (A) w1118 + DMSO, 1 hour, nerve severed, (B) w1118 + DMSO, 1 hour, nerve intact, (C) w1118 + 500 μM rotenone (ROT), 1 hour, nerve severed, (D) w1118 + ROT, 1 hour, nerve intact, (E) w1118 + DMSO, 2 hours, nerve severed, (F) w1118 + DMSO, 2 hours, nerve intact, (G) (w1118 + 500 μM ROT, 2 hours, nerve severed), (H) (w1118 + ROT, 2 hours, nerve intact), (I) (w1118 + DMSO, 4 hours, nerve severed), (J) (w1118 + DMSO, 4 hours, nerve intact), (K) (w1118 + 500 μM ROT, 4 hours, nerve severed), (L) (w1118 + ROT, 4 hours, nerve intact), (M) (w1118 + DMSO, 6 hours, nerve severed), (N) (w1118 + DMSO, 6 hours, nerve intact), (O) (w1118 + 500 μM ROT, 6 hours, nerve severed), (P) (w1118 + 500 μM ROT, 6 hours, nerve intact), (Q) (w1118 + DMSO, 8 hours feeding, intact larvae), (R) (w1118 + 25 μM ROT, 8 hours feeding, intact larvae), (S) (w1118 + DMSO, embryo to 3rd instar), and (T) (w1118 + 25 μM ROT, embryo to 3rd instar) larvae immunostained with antibodies against the active zone scaffold Bruchpilot (BRP:fire-LuT) to label the active zones. Scale bar: 5 μm. Note that the incubation or feeding larvae with rotenone elevate BRP levels at the NMJs in w1118 + 500 μM ROT, 6 hours nerve intact and w1118 + 25 μM ROT, embryo to 3rd instar compared to control nerve severed larvae. (U) Histograms show the quantification of BRP intensity in μm2 of bouton area at muscle 6/7 in the indicated genotypes. At least 8 NMJs of each genotype were used for quantification. ***p = 0.0003 (w1118 + DMSO, 1 hour, nerve severed vs. w1118 + DMSO, 1 hour, nerve intact), ***p < 0.0001, **p = 0.0019 (w1118 + DMSO, embryo to 3rd instar vs. w1118 + 25 μM ROT, embryo to 3rd instar). Error bars signify the standard error of the mean. Statistical analysis is based on the Student’s t test for pairwise comparison among the samples. Raw data for this figure are available in the S2 Data Excel file, tab S12 Fig. (TIF) [file pbio.3003388.s015.tif]

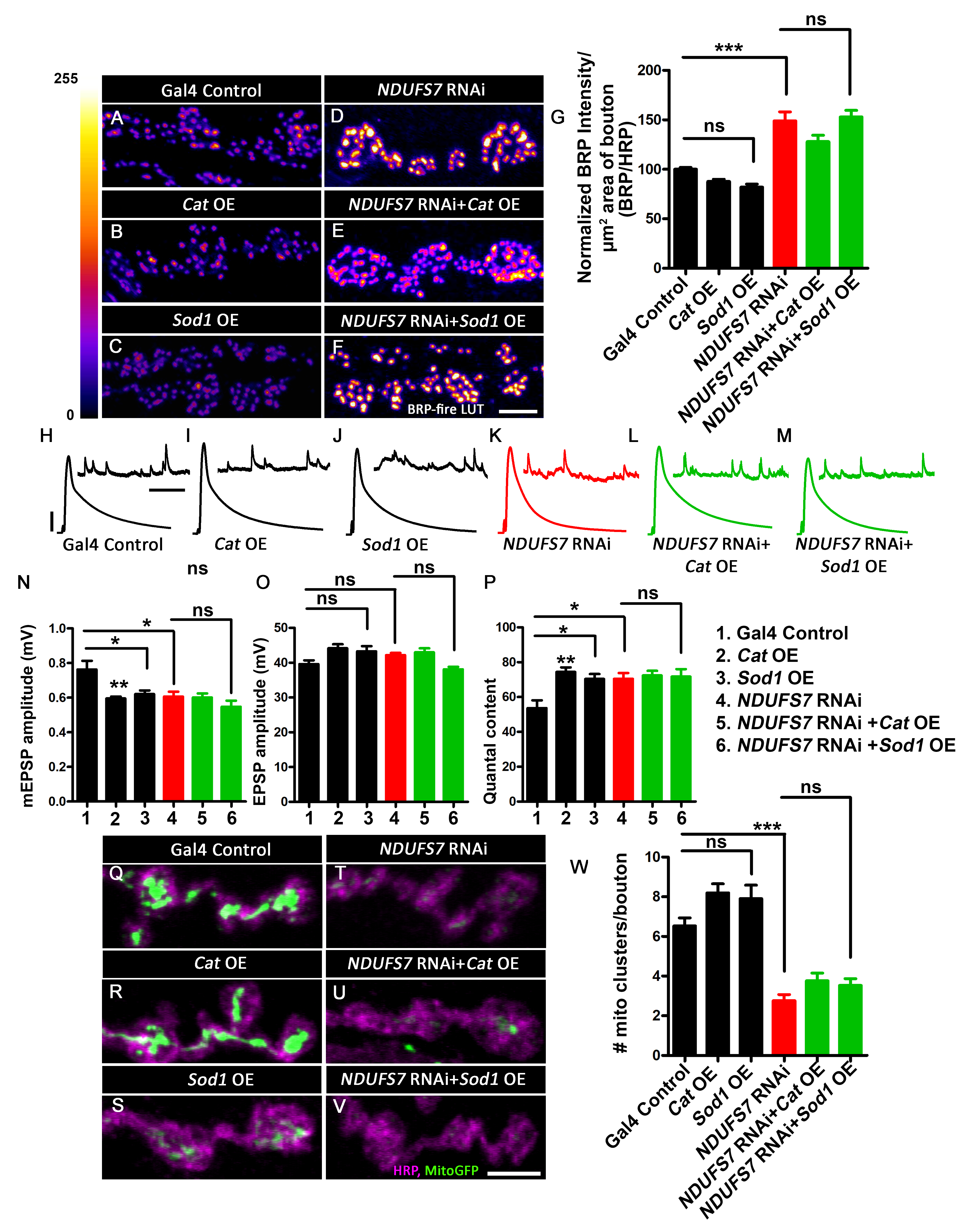

Supplement: S13 Fig — Representative images of the A2 hemisegment of muscle 6/7 NMJs in (A) UAS-mitoGFP, D42-Gal4/+, (B) UAS-Cat/+; UAS-mitoGFP, D42-Gal4/+, (C) UAS-Sod1/+; UAS-mitoGFP, D42-Gal4/+, (D) NDUFS7[RNAi]/+; UAS-mitoGFP, D42-Gal4/+, (E) NDUFS7[RNAi]/UAS-Cat; UAS-mitoGFP, D42-Gal4/+, and (F) NDUFS7[RNAi]/UAS-Sod1; UAS-mitoGFP, D42-Gal4/+ larvae immunostained with antibodies against the active zone scaffold Bruchpilot (BRP:fire-LuT) to label the active zones. BRP levels are upregulated at the NMJs in NDUFS7-depleted flies, while overexpression of ROS scavenger genes Cat or Sod1 in the neuron fails to restore BRP to the control level. (A–F) Scale bar: 2.5 μm. (F and G) Histograms showing quantification of (F) BRP intensity and (G) density per μm2 area of bouton at muscle 6/7 for the genotypes mentioned above. At least 8 NMJs of each genotype were used for quantification. ***p < 0.0001. Error bars denote mean ± s.e.m. Statistical analysis based on one-way ANOVA followed by post-hoc Tukey’s multiple-comparison test. (H–P) Representative electrophysiological traces and quantifications of mEPSPs, EPSPs and quantal content in the indicated genotypes. Scale bars for EPSPs (mEPSP) are x = 50 ms (1,000 ms) and y = 10 mV (1 mV). EPSP amplitudes were maintained in NDUFS7-depleted flies, likely due to the induction of BRP. A minimum of 7 NMJs recordings of each genotype were used for quantification. mEPSP amplitude: **p = 0.002 (Control versus UAS-Cat), *p = 0.009 (Control versus UAS-Sod1), *p = 0.018 (Control versus NDUFS7[RNAi]); Quantal content: **p = 0.0009 (Control versus UAS-Cat), *p = 0.004 (Control versus UAS-Sod1), *p = 0.010 (Control versus NDUFS7[RNAi]); ns, not significant. Statistical analysis based on one-way ANOVA followed by post-hoc Tukey’s multiple-comparison test. Error bars denote the standard error of the mean. (Q) Representative images of the A2 hemisegment of muscle 6/7 NMJs in UAS-mitoGFP, D42-Gal4/+, (R) UAS-Cat/+; UAS-mitoGFP, D42-Gal4/+, (S) UAS-Sod1/+; UAS-mitoGFP [file pbio.3003388.s016.tif]

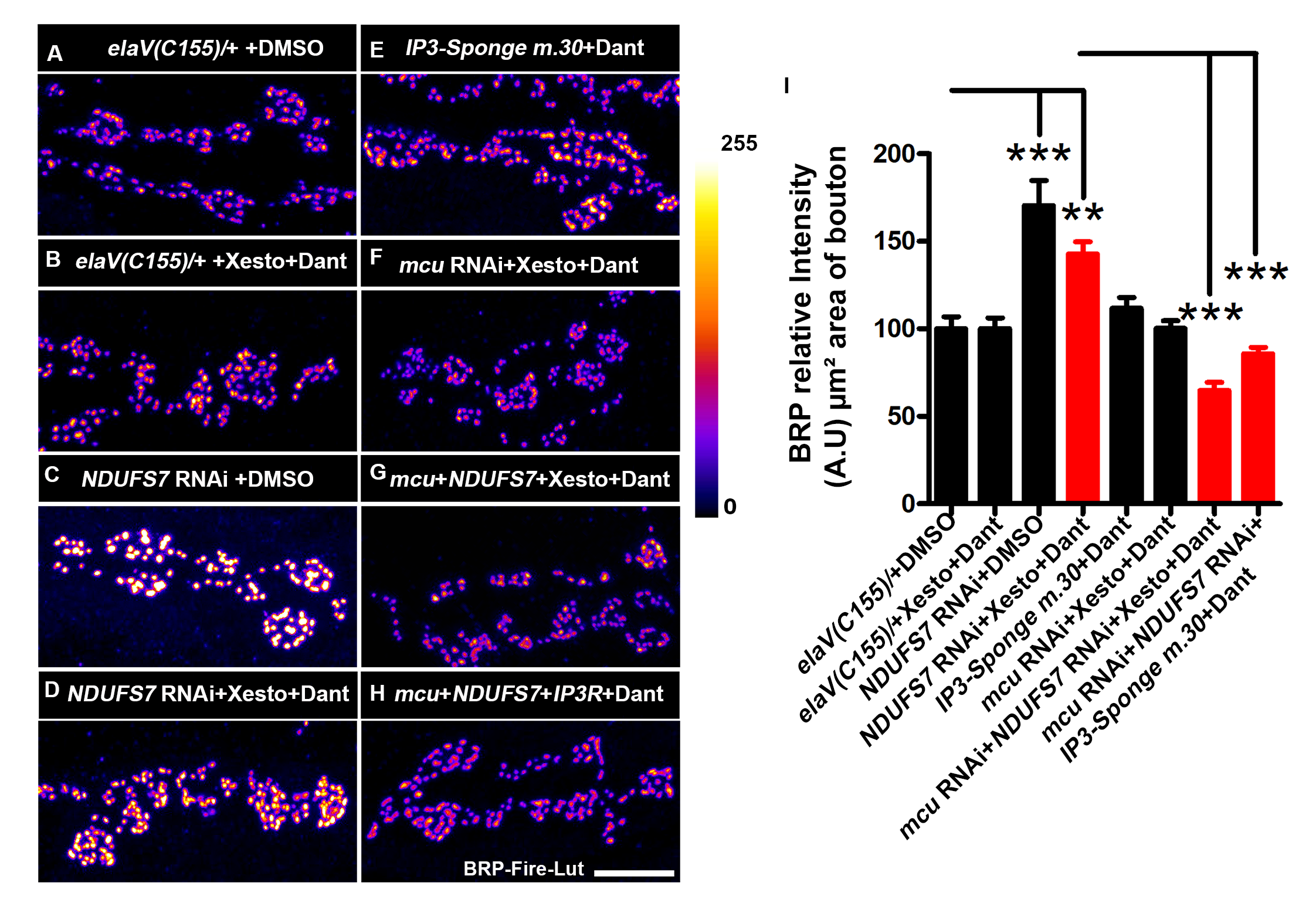

Supplement: S14 Fig — Representative images of the A2 hemisegment of muscle 6/7 NMJs in (A) elaV(C155)/+ with DMSO, (B) elaV(C155)/+ with Xestospongin C (Xesto) and Dantrolene (Dant), (C) elaV(C155)/+ ; UAS-NDUFS7[RNAi]/+ with DMSO, (D) elaV(C155)/ + ; UAS-NDUFS7[RNAi]/+ with Xesto and Dant, (E) elaV(C155)/+; UAS-IP3-sponge.m30/+, (F) elaV(C155)/+; UAS-mcu[RNAi]/+, (G) elaV(C155)/+; UAS-mcu[RNAi]/UAS-NDUFS7[RNAi] with Xesto and Dant and (H) elaV(C155)/+; UAS-mcu[RNAi]/UAS-NDUFS7[RNAi]/+; UAS-IP3-sponge.m30/+ with Dant. Larvae were immunostained with antibodies against the active zone scaffold Bruchpilot (BRP:fire-LuT) to label the active zones. BRP levels are upregulated at the NMJs in UAS-NDUFS7[RNAi] with DMSO and UAS-NDUFS7[RNAi] with Xesto and Dant flies. However, genetically inhibiting ER calcium release and calcium import to the mitochondria reduces BRP levels below the control level. (A–H) Scale bar: 5 μm. (I) Histograms showing quantification of BRP intensity in the μm2 area of bouton at muscle 6/7 in the above genotypes. At least 8 NMJs of each genotype were used for quantification. ***p < 0.0001 **p = 0.0002, (BRP levels: elaV(C155)/+ with DMSO vs. elaV(C155)/+; UAS-NDUFS7[RNAi]/+ with Xesto and Dant, Error bars denote mean ± s.e.m. Statistical analysis based on one-way ANOVA followed by post-hoc Tukey’s multiple-comparison test. Raw data for this figure are available in the S2 Data Excel file, tab S14 Fig. (TIF) [file pbio.3003388.s017.tif]

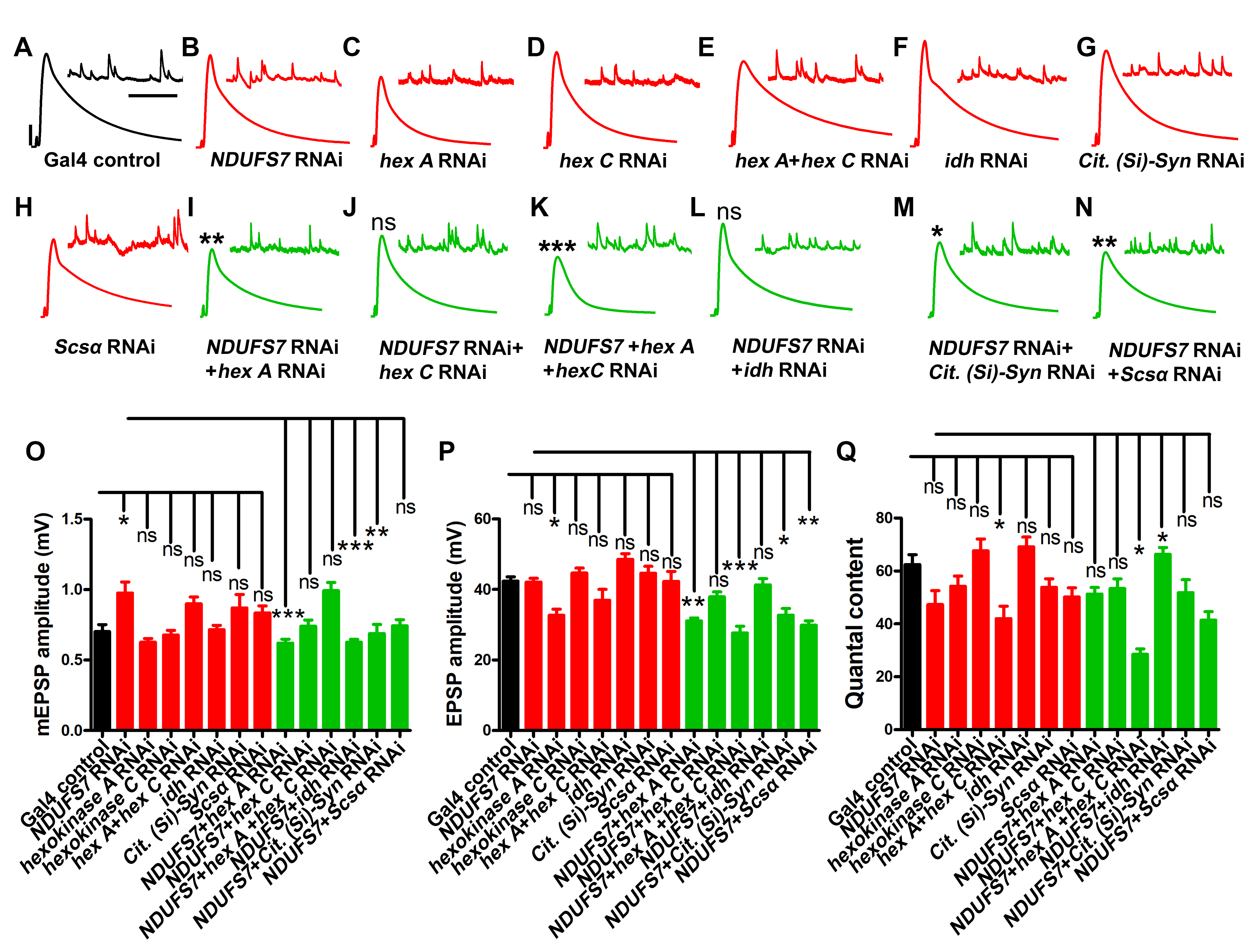

Supplement: S15 Fig — (A–N) Representative electrophysiological traces of Gal4 driver control (elaV(C155)-Gal4/+) or experimental genotypes tested for combinatorial effects when losing NDUFS7 gene function and/or the function of glycolysis or TCA cycle genes, including: hexokinase A (hex-A), hexokinase C (hex-C), Citrate (Si) Synthase I, Isocitrate dehydrogenase (Idh), and Succinyl-coenzyme A synthetase α subunit 1 (Scsa1). Scale bars for EPSPs (mEPSP) are x = 50 ms (1,000 ms) and y = 10 mV (1 mV). (O–Q) Data histograms and statistical analyses for these same genotypes (knockdowns all in the elaV(C155)-Gal4/ + genetic background; see Table Q in the S1 Tables file for full genotypes), including quantal size (mEPSP), evoked excitation (EPSP) and quantal content. Error bars denote mean ± s.e.m. Statistical analysis based on one-way ANOVA followed by post-hoc Tukey’s multiple-comparison test. *p < 0.05, **p < 0.01, ***p < 0.001. Raw data for this figure are available in the S2 Data Excel file, tab S15 Fig. (TIF) [file pbio.3003388.s018.tif]

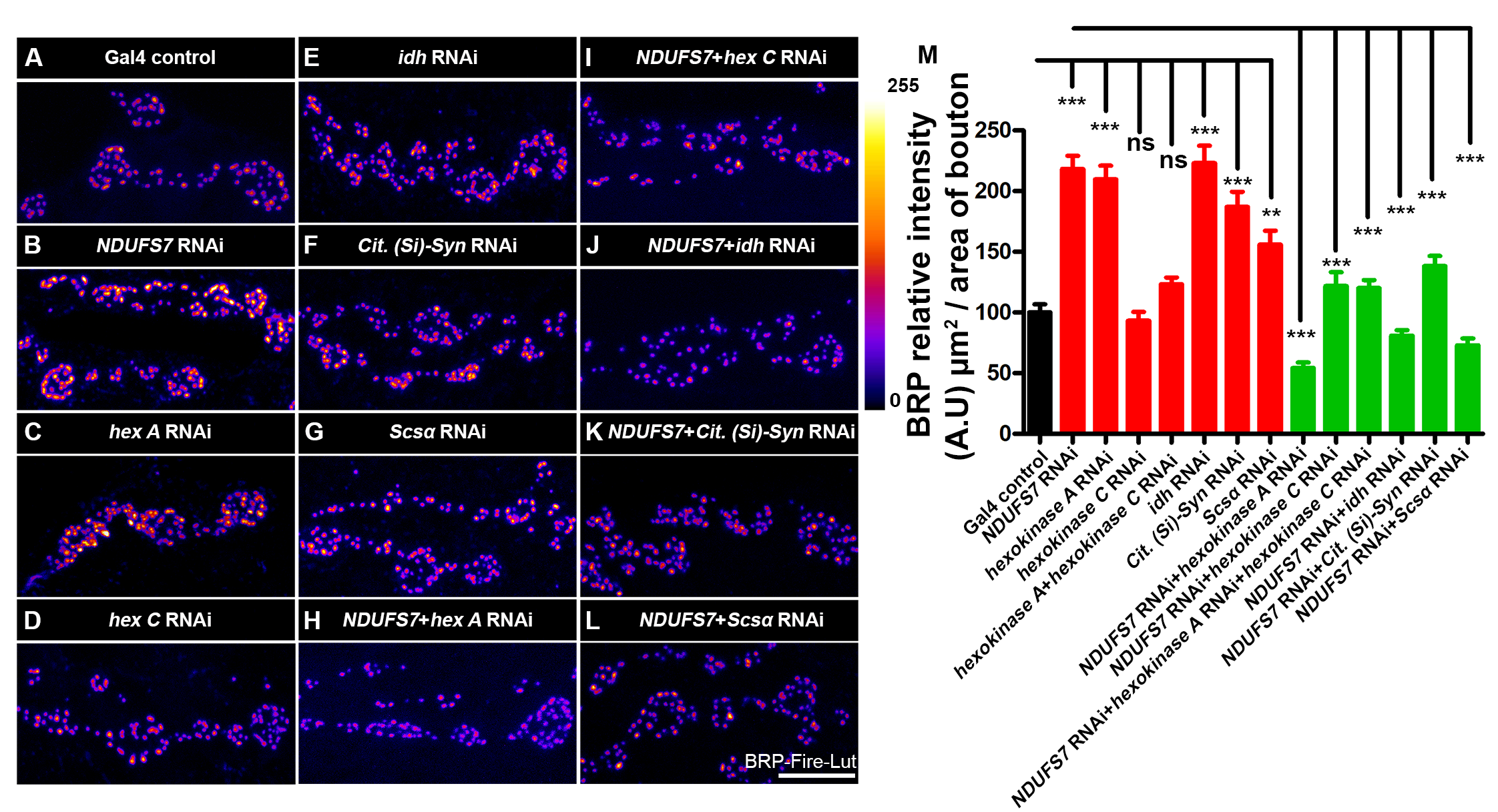

Supplement: S16 Fig — (A–L) Representative images of the A2 hemisegment of muscle 6/7 NMJs, examining active zone material through anti-Bruchpilot (BRP) immunostaining. Analysis for Gal4 driver control (elaV(C155)-Gal4/+) or experimental genotypes tested for combinatorial effects of losing NDUFS7 gene function and/or the function of glycolysis or TCA cycle genes, including hexokinase A (hex-A), hexokinase C (hex-C), Citrate (Si) Synthase I, Isocitrate dehydrogenase (Idh), and Succinyl-coenzyme A synthetase α subunit 1 (Scsa1). Scale bar: 5 μm. See Table R in the S1 Tables file for full genotypes. (M) Histograms showing quantification of BRP intensity in the μm2 area of bouton at muscle 6/7 in the above genotypes. At least 8 NMJs of each genotype were used for quantification. Error bars signify mean ± s.e.m. Raw data for this figure are available in the S2 Data Excel file, tab S16 Fig. (TIF) [file pbio.3003388.s019.tif]

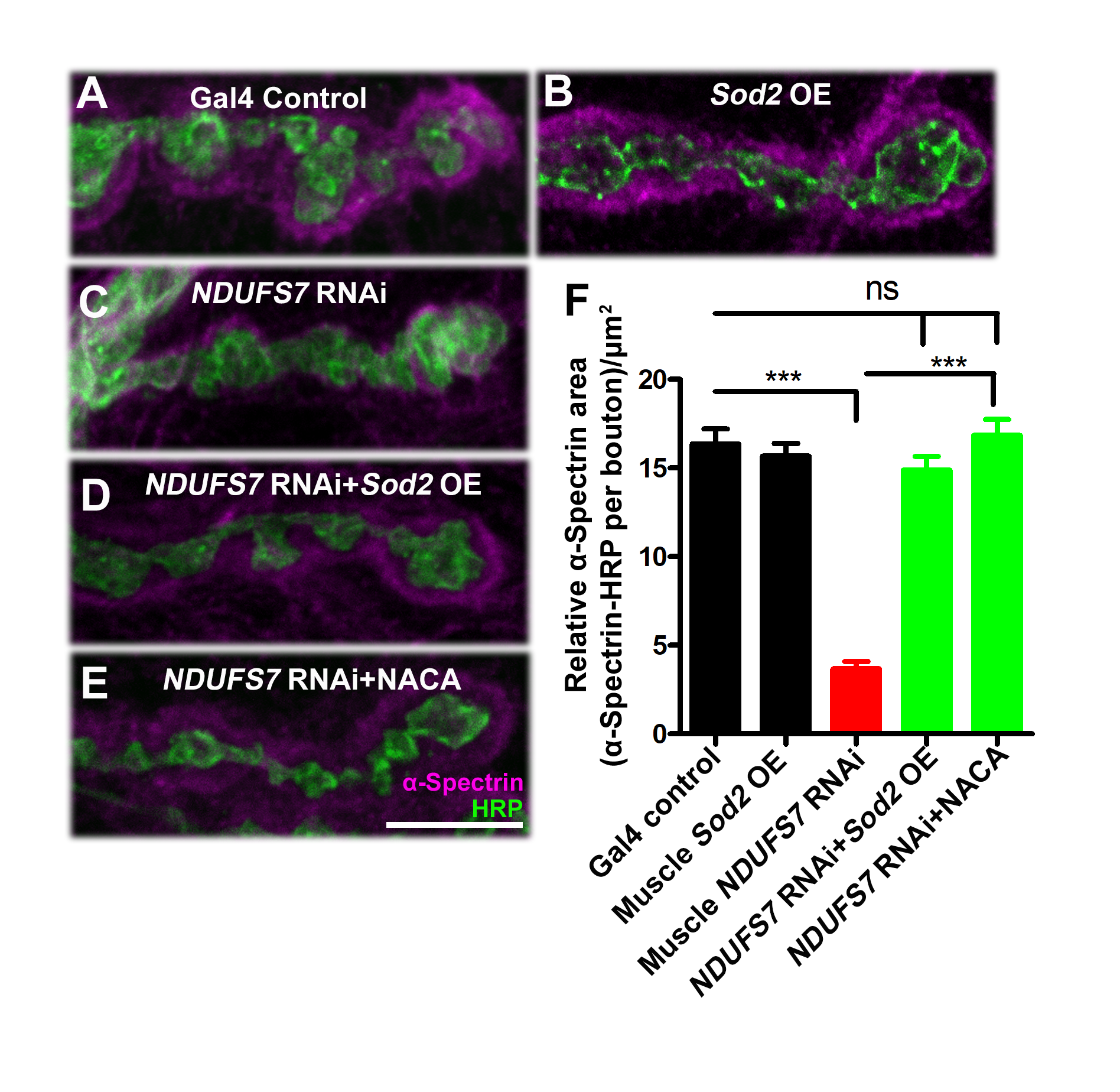

Supplement: S17 Fig — (A–E) Representative confocal images of third instar larval NMJs in (A) Muscle-Gal4 control (BG57-Gal4/+), (B) Muscle Gal4-driven UAS-Sod2 (UAS-Sod2/+; BG57-Gal4/+), (C) Muscle UAS-NDUFS7[RNAi] (UAS-NDUFS7[RNAi]/+; BG57-Gal4/+), (D) Sod2 muscle rescue (UAS-Sod2/UAS-NDUFS7[RNAi]; BG57-Gal4/BG57-Gal4), and (E) (UAS-NDUFS7[RNAi]/+; BG57-Gal4/+ with NACA). Synapses are immunostained with anti-HRP (green) and α-Spectrin (magenta) antibodies. Scale bar: 5 μm. (N) Histogram showing relative α-Spectrin area in the indicated genotypes. Compared with controls, UAS-NDUFS7[RNAi]/ + ; BG57-Gal4/+) NMJs show a significant reduction in α-Spectrin area, which is restored upon muscle overexpression of a Sod2 transgene or feeding larvae with NACA. ***p < 0.0001; ns, not significant. Error bars signify mean ± s.e.m. Statistical analysis based on one-way ANOVA with post-hoc Tukey’s test for multiple comparisons. Raw data for this figure are available in the S2 Data Excel file, tab S17 Fig. (TIF) [file pbio.3003388.s020.tif]

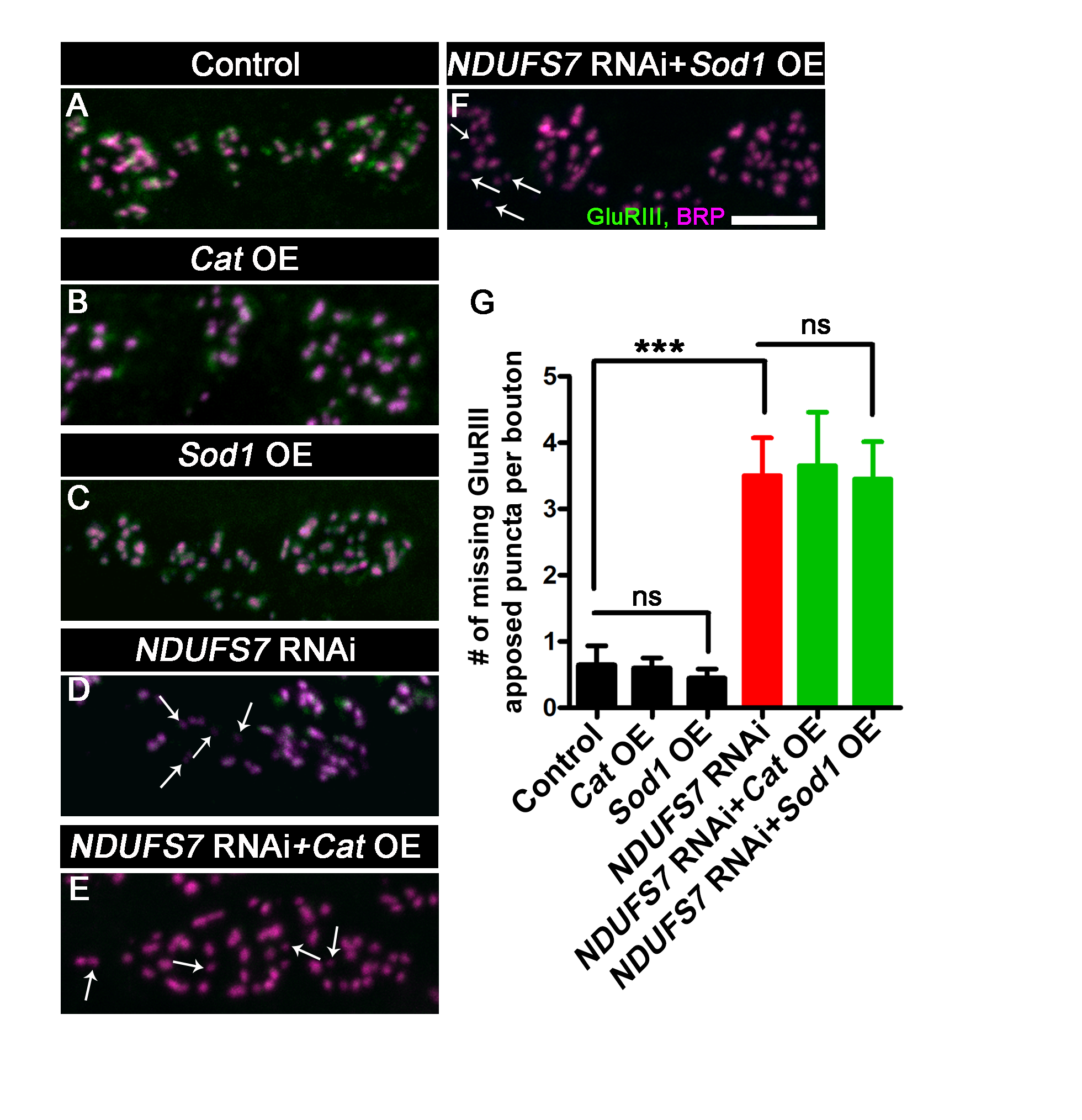

Supplement: S18 Fig — The NDUFS7 subunit in muscle affects the organization of the GluR cluster in Drosophila. Representative confocal images of boutons at third instar larval NMJ synapse in (A) Muscle-Gal4 control (BG57-Gal4/+), (B) Muscle Gal4-driven UAS-Cat (UAS-Cat/+; BG57-Gal4/+), (C) Muscle Gal4-driven UAS-Sod1 (UAS-Sod1/+; BG57-Gal4/+), (D) Muscle NDUFS7[RNAi] (NDUFS7[RNAi]/+; BG57-Gal4/+), (E) Cat muscle rescue (UAS-Cat/NDUFS7[RNAi]; BG57-Gal4/BG57-Gal4), and (F) Sod1 muscle rescue (UAS-Sod1/NDUFS7[RNAi]; BG57-Gal4/BG57-Gal4) animals immunolabeled with active zone marker BRP (magenta) and anti-GluRIII (green) antibodies. Scale bar: 2.5 μm. Note that GluRIII clusters apposed by BRP are missing in the NDUFS7-depleted NMJs, as well as the Cat and Sod1 non-rescued NMJs (marked in arrow). (G) Histograms showing quantification of the number of missing BRP-GluRIII apposed puncta per bouton in the indicated genotypes. ***p < 0.0001. Error bars represent mean ± s.e.m. Statistical analysis based on one-way ANOVA with post-hoc Tukey’s test for multiple comparisons. Raw data for this figure are available in the S2 Data Excel file, tab S18 Fig. (TIF) [file pbio.3003388.s021.tif]
