# Supplementary material for: Mitochondrial Complex I and ROS control neuromuscular function through opposing pre- and postsynaptic mechanisms
Source: PLoS Biol. 2025 Sep 22;23(9):e3003388. doi: 10.1371/journal.pbio.3003388 (PMC12478897; doi:10.1371/journal.pbio.3003388)
Supplement: S1 Tables — Tables A–U: summary data for graphs in all figures and supplemental figures. Table V: detailed information about reagents and materials for items described in the STAR methods section. (PDF) [file pbio.3003388.s001.pdf]

Mitochondrial Complex I and ROS control neuromuscular function through opposing pre- and postsynaptic mechanisms

This is the S1 Tables file for Mallik *et al*. It contains the following:

- Tables A-U: Summary data for graphs in all Main Figures and Supplemental Figures.
- Table V: Detailed information about reagents and materials for items described in the STAR METHODS section.

Table A, related to Fig 2: Mitochondrial branch length (μm) and cluster number (μm^2 area of the axon).

**Genotype**	**Mitochondria branch length (μm) in VNC**	**# clusters (per μm^2 area of a nerve at A5 hemisegment)**
UAS-mitoGFP, D42-Gal4/+	1.74 ± 0.07, n = 50	0.79 ± 0.03, n = 30
UAS-drp1[RNAi]/+; UAS-mitoGFP, D42-Gal4/+	1.69 ± 0.08, n = 50	0.80 ± 0.02, n = 30
UAS-NDUFS7[RNAi]/+; UAS-mitoGFP, D42-Gal4/+	0.96 ± 0.04, n = 50	0.27 ± 0.01, n = 30
UAS-mitoGFP, D42-Gal4/UAS-marf[RNAi]	0.89 ± 0.03, n = 50	0.20 ± 0.02, n = 30
UAS-NDUFS7[RNAi]/+; UAS-mitoGFP, D42-Gal4/UAS-marf[RNAi]	0.83 ± 0.03, n = 50	0.20 ± 0.01, n = 30
UAS-NDUFS7 [RNAi]/UAS-drp1[RNAi]; UAS-mitoGFP, D42-Gal4/+	0.91 ± 0.03, n = 50	0.32 ± 0.02, n = 30

D42-Gal4 is a motor neuron driver. Values represent mean ± s.e.m.

Table B, related to S4 Fig: mitochondrial branch length (μm) and cluster number (per μm^2 area of the axon).

**Genotype**	**Mito branch length (μm) in VNC**	**# clusters (per μm^2 area of nerve A5 hemi segment)**
D42-Gal4, UAS-mitoGFP/+	1.88 ± 0.07, n = 50	0.94 ± 0.05, n = 30
UAS-Sod2/+; D42-Gal4, UAS-mitoGFP/+	1.75 ± 0.06, n = 50	1.10 ± 0.06, n = 35
UAS-NDUFS7 [RNAi]/+; D42-Gal4, UAS-mitoGFP/+	0.99 ± 0.03, n = 50	0.21 ± 0.01, n = 30
UAS-NDUFS7 [RNAi]/+; D42-Gal4,UAS-mitoGFP/+ with NACA	1.63 ± 0.06, n = 50	0.59 ± 0.04, n = 30
UAS-NDUFS7 [RNAi]/UAS-Sod2; D42-Gal4,UAS-mitoGFP/+	1.94 ± 0.08, n = 50	0.57 ± 0.03, n = 30
UAS-ND-30[RNAi]/+; *D42-Gal4, UAS-mitoGFP/+*	0.89 ± 0.03, n = 50	0.32 ± 0.01, n = 30
UAS-Sod2/*UAS-ND-30[RNAi]*; *D42-Gal4, UAS-mitoGFP/+*	1.78 ± 0.06, n = 50	0.83 ± 0.04, n = 30

D42-Gal4 is a motor neuron driver. Values represent mean ± s.e.m.

Table C, related to S5 Fig: mitochondrial branch length (µm) and cluster number (per $µm^2$ area of the axon).

Genotype	Mitochondrial branch length (µm) in VNC	# clusters (per $µm^2$ area of axon at A5 hemi segment)
UAS-mitoGFP, D42-Gal4/+	1.21 ± 0.05, n=40	1.43 ± 0.06, n=30
UAS-Cat/+; UAS-mitoGFP, D42-Gal4/+	1.22 ± 0.05, n=40	1.50 ± 0.09, n=30
UAS-Sod1/+; UAS-mitoGFP, D42-Gal4/+	1.25 ± 0.03, n=40	1.51 ± 0.11, n=30
UAS-NDUFS7[RNAi]/+; UAS-mitoGFP, D42-Gal4/+	0.72 ± 0.03, n=40	0.68 ± 0.04, n=30
UAS-NDUFS7[RNAi]/UAS-Cat; UAS-mitoGFP, D42-Gal4/+	0.65 ± 0.02, n=40	0.54 ± 0.05, n=30
UAS-NDUFS7[RNAi]/UAS-Sod1; UAS-mitoGFP, D42-Gal4/+	0.69 ± 0.02, n=40	0.66 ± 0.04, n=30

D42-Gal4 is a motor neuron driver. Values represent mean ± s.e.m.

Table D, related to Fig 2: mitochondrial cluster number (per μm^2 area of bouton).

Genotype	# clusters (per μm^2 area of bouton)
UAS-mitoGFP, D42-Gal4/+	4.67 ± 0.26, n = 40
UAS-Sod2/+; UAS-mitoGFP, D42-Gal4/+	5.2 ± 0.20, n = 40
UAS-NDUFS7[RNAi]/+; UAS-mitoGFP, D42-Gal4/+	1.40 ± 0.15, n = 40
UAS-NDUFS7[RNAi]/UAS-Sod2; UAS-mitoGFP, D42-Gal4/+	2.07 ± 0.15, n = 40
UAS-drp1[RNAi]/+; UAS-mitoGFP, D42-Gal4/+	4.65 ± 0.24, n = 40
UAS-drp1[RNAi]/UAS-Sod2; UAS-mitoGFP, D42-Gal4/+	5.47 ± 0.22, n = 40
UAS-drp1[RNAi]/UAS-NDUFS7[RNAi]; UAS-mitoGFP, D42-Gal4/+	1.15 ± 0.19, n = 40
UAS-mitoGFP, D42-Gal4/UAS-marf[RNAi]	1.50 ± 0.22, n = 40
UAS-Sod2/+; UAS-mitoGFP, D42-Gal4/UAS-marf[RNAi]	2.40 ± 0.19, n = 40
UAS-NDUFS7[RNAi]/+; UAS-mitoGFP, D42-Gal4/UAS-marf[RNAi]	0.62 ± 0.13, n = 40
UAS-NDUFS7[RNAi]/UAS-Sod2; UAS-mitoGFP, D42-Gal4/UAS-marf[RNAi]	0.92 ± 0.14, n = 40

D42-Gal4 is a motor neuron driver. Values represent mean ± s.e.m.

Table E, related to S2 Fig and S6 Fig: MitoSOX intensity as an indicator of mitochondrial ROS.

**Genotype**	**MitoSOX Intensity in AU (μm^2 area of indicated tissue)**
**Ventral nerve cord (VNC)** (MCI genetic manipulation)	
D42-Gal4, UAS-mitoGFP/+	100.0 ± 4.47, n = 30
UAS-NDUFS7[RNAi]/+;<br>*D42-Gal4, UAS-mitoGFP/+*	352.1 ± 8.44, n = 30
UAS-NDUFS7[RNAi]/+;<br>*D42-Gal4, UAS-mitoGFP/+* with NACA	83.50 ± 5.18, n = 30
UAS-NDUFS7[RNAi]/UAS-Sod2;<br>*UAS-mitoGFP, D42-Gal4/+*	94.21 ± 6.98, n = 30
**Muscle**	
UAS-mitoGFP/+; BG57-Gal4/+	100.0 ± 8.12, n = 30
UAS-NDUFS7[RNAi]/UAS-mitoGFP;<br>*BG57-Gal4/+*	267.6 ± 14.62, n = 30
UAS-NDUFS7[RNAi]/UAS-mitoGFP;<br>*BG57-Gal4/+* with NACA	93.46 ± 8.10, n = 30
UAS-NDUFS7[RNAi]/UAS-Sod2; BG57-Gal4/+	92.34 ± 5.31, n = 30
**Ventral nerve cord (VNC)** (Mitofusin or pharmacology against MCI)	
D42-Gal4, UAS-mitoGFP/+	100.0 ± 13.84, n = 30
D42-Gal4, UAS-mitoGFP/UAS-marf[RNAi]	1067 ± 91.59, n = 37
D42-Gal4, UAS-mitoGFP/+ with 25μM ROT	405.3 ± 40.72, n = 22
UAS-NDUFS7[RNAi]/+;<br>*D42-Gal4, UAS-mitoGFP/+*	645.5 ± 37.13, n = 47
**Axon**	
D42-Gal4, UAS- mitoGFP/+	100.0 ± 12.28, n = 30
D42-Gal4,UAS-mitoGFP/UAS-marf[RNAi]	325.9 ± 23.02, n = 48
D42-Gal4,UAS- mitoGFP/+ with 25μM ROT	490.0 ± 52.21, n = 40
UAS-NDUFS7[RNAi]/+;<br>*D42-Gal4, UAS-mitoGFP/+*	919.6 ± 66.61, n = 65
**Bouton**	
D42-Gal4, UAS-mitoGFP/+	100.0 ± 10.49, n = 50
D42-Gal4,UAS-mitoGFP/UAS-marf[RNAi]	254.7 ± 46.29, n = 53
D42-Gal4,UAS- mitoGFP/+ with 25μM ROT	271.5 ± 24.21, n = 56
NDUFS7 [RNAi]/+; D42-Gal4, UAS-mitoGFP/+	223.2 ± 19.16, n = 59

NACA – N-Acetyl Cysteine Amide; ROT – Rotenone. *D42-Gal4* is a motor neuron driver. *BG57-Gal4* is a muscle driver. Values represent mean ± s.e.m.

Table F, related to S3 Fig: MitoSOX intensity as an indicator of mitochondrial ROS for genetic combinations, as analyzed in the ventral nerve cord, muscle, axon, or boutons.

**Genotype**	**MitoSOX Intensity in AU (μm^2 area of VNC, axon, boutons and muscle**
**Ventral nerve cord (VNC)**	
UAS-mitoGFP, D42-Gal4/+	100.0 ± 9.98, n=20
UAS-mitoGFP, D42-Gal4/UAS-Sod2[RNAi]	175.6 ± 15.52, n=22
**Muscle**	
UAS-mitoGFP/+; BG57-Gal4/+	100.0 ± 6.01, n=41
UAS-mitoGFP/+; BG57-Gal4/UAS-Sod2[RNAi]	145.5 ± 9.42, n=40
**Axon**	
UAS-mitoGFP, D42-Gal4/+	100.0 ± 6.99, n=28
UAS-mitoGFP, D42-Gal4/UAS-Sod2[RNAi]	143.4 ± 17.07, n=27
**Bouton**	
UAS-mitoGFP, D42-Gal4/+	100.0 ± 10.56, n=25
UAS-mitoGFP, D42-Gal4/UAS-Sod2[RNAi]	266.8 ± 14.67, n=25

D42-Gal4 is a motor neuron driver. *BG57-Gal4* is a muscle driver. Values represent mean ± s.e.m.

Table G, related to Fig 3, Fig 8, S7 Fig, and S17 Fig: Cell and synapse-level phenotypes from tissue-specific losses of MCI.

Genotype	% of A5 axons showing CSP accumulation	% Futsch positive loops	Genotype	α-Spectrin levels (AU)	# missing apposed BRP-puncta (per bouton)
D42-Gal4/+	12.50 ± 12.50, n = 8	36.47 ± 2.88, n = 8	*BG57-Gal4/+*	16.35 ± 0.84, n = 30	GluRIII: 0.75 ± 0.23, n = 20<br>GluRIIA: 1.09 ± 0.23, n = 21
UAS-Sod2/+; D42-Gal4/+	16.67 ± 16.67, n = 6	50.72 ± 3.95, n = 8	*NDUFS7 [RNAi]/+; BG57-Gal4/+*	15.67 ± 0.72, n = 30	GluRIII: 3.50 ± 0.62, n = 20<br>GluRIIA: 4.19 ± 0.62, n = 21
UAS-NDUFS7 [RNAi]/+; D42-Gal4/+	87.50 ± 12.50, n = 8	21.54 ± 1.97, n = 8	*UAS-Sod2/+; BG57-Gal4/+*	3.67 ± 0.41, n = 30	GluRIII: 0.30 ± 0.12, n = 20<br>GluRIIA: 0.66 ± 0.21, n = 21
UAS-NDUFS7 [RNAi]/+; D42-Gal4/+ with NACA	16.67 ± 16.67, n = 6	40.52 ± 4.36, n = 8	*NDUFS7 [RNAi]/+;BG57-Gal4/+* with NACA	14.89 ± 0.77, n = 30	GluRIII: 0.50 ± 0.22, n = 20<br>GluRIIA: 0.52 ± 0.13, n = 21
NDUFS7 [RNAi]/ UAS-Sod2; D42-Gal4/+	16.67 ± 11.24, n = 12	38.97 ± 3.34, n = 8	*UAS-Sod2/NDUFS7 [RNAi]; BG57-Gal4/BG57-Gal4*	16.84 ± 0.91, n = 30	GluRIII: 0.40 ± 0.13, n = 20<br>GluRIIA: 0.61 ± 0.20, n = 21

For presynaptic genotypes (left): % of A5 axons showing accumulation of CSP, % of Futsch positive loops. For postsynaptic genotypes: α-Spectrin levels and number of missing BRP puncta apposed to glutamate receptor cluster (GluRIII is also known as GluRIIC). *D42-Gal4* is a motor neuron driver. *BG57-Gal4* is a muscle driver. Values represent mean ± s.e.m.

Table H, related to S8 Fig, S9 Fig, and S18 Fig: Cell and synapse-level phenotypes from tissue-specific loss of MCI.

Genotype	% of A5 axon showing CSP accumulation	% Futsch positive loops	Genotype	# missing BRP-GluRIII apposed puncta per bouton
UAS-mitoGFP, D42-Gal4/+	Normal: 88.89 ± 12.50, n=9<br>Accumulation: 11.11 ± 11.11, n=9	68.75 ± 3.13, n=14	*BG57-Gal4/+*	GluRIII: 0.65 ± 0.28, n=20
UAS-Cat/+; UAS-mitoGFP,D42-Gal4	Normal: 83.89 ± 11.11, n=9<br>Accumulation: 11.11 ± 11.11, n=9	70.93 ± 3.76, n=10	*UAS-Cat/+; BG57-Gal4/+*	GluRIII: 0.60 ± 0.15, n=20
UAS-Sod1/+; UAS-mitoGFP,D42-Gal4	Normal: 92.31 ± 7.69, n=13<br>Accumulation: 7.69 ± 7.69, n=13	71.74 ± 2.51, n=9	*UAS-Sod1/+; BG57-Gal4/+*	GluRIII: 0.45 ± 0.13, n=20
NDUFS7 [RNAi]/+; UAS-mitoGFP,D42-Gal4/+	Normal: 22.22 ± 14.70, n=9<br>Accumulation: 77.78 ± 14.70, n=9	33.03 ± 2.48, n=10	*NDUFS7 [RNAi]/+; BG57-Gal4/+*	GluRIII: 3.50 ± 0.57, n=20
NDUFS7 [RNAi]/UAS-Cat; UAS-mitoGFP, D42-Gal4/+	Normal: 20.00 ± 13.33, n=10<br>Accumulation: 80.00 ± 13.33, n=10	40.32 ± 2.85, n=10	*NDUFS7 [RNAi]/UAS-Cat; BG57-Gal4/BG57-Gal4*	GluRIII: 3.65 ± 0.80, n=20
NDUFS7 [RNAi]/UAS-Sod1; UAS-mitoGFP,D42-Gal4/+	Normal: 22.22 ± 14.70, n=9<br>Accumulation: 77.78 ± 14.70, n=9	39.88 ± 1.84, n=10	*NDUFS7 RNAi/UAS-Sod1; BG57-Gal4/BG57-Gal4*	GluRIII: 3.45 ± 0.56, n=20

For presynaptic genotypes (left): % of A5 axons showing accumulation of CSP, % of Futsch positive loops. For postsynaptic genotypes: Number of missing BRP puncta apposed to glutamate receptor cluster (GluRIII is also known as GluRIIC. *D42-Gal4* is a motor neuron driver. *BG57-Gal4* is a muscle driver. Values represent mean ± s.e.m.

Table I, related to S10 Fig: Electrophysiological parameters when NDUFS7 and Mitofusin (*marf*) functions are impaired – mEPSPs, EPSPs, mEPSP frequencies, and QC.

**Genotype**	**mEPSP amp (mV)**	**mEPSP freq (Hz)**	**EPSP amplitude (mV)**	**Quantal content (QC)**
UAS-mitoGFP; D42-Gal4/+	1.06 ± 0.05, n = 10	4.12 ± 0.72, n = 10	49.27 ± 0.99, n = 10	47.30 ± 2.49, n = 10
UAS-Sod2/+; UAS-mitoGFP, D42-Gal4/+	0.86 ± 0.08, n = 9	2.77 ± 0.51, n = 9	43.53 ± 2.55, n = 10	53.16 ± 4.43, n = 9
NDUFS7 [RNAi]/+; UAS-mitoGFP, D42-Gal4/+	0.79 ± 0.02, n = 13	3.30 ± 0.41, n = 13	45.49 ± 1.18, n = 13	57.79 ± 2.39, n = 13
UAS-mitoGFP, D42-Gal4/UAS-marf[RNAi]	0.63 ± 0.05, n = 11	4.99 ± 0.76, n = 11	39.68 ± 1.39, n = 12	65.35 ± 5.30, n = 11
UAS-Sod2/+; UAS-mitoGFP, D42-Gal4/UAS-marf[RNAi]	0.83 ± 0.06, n = 8	5.01 ± 0.74, n = 8	37.45 ± 2.08, n = 9	47.52 ± 4.55, n = 8
UAS- NDUFS7 [RNAi]/+; UAS- mitoGFP, D42-Gal4/UAS-marf[RNAi]	0.90 ± 0.07, n = 10	6.23 ± 0.66, n = 10	35.44 ± 1.80, n = 12	40.82 ± 3.70, n = 10
UAS-Sod2/UAS-NDUFS7 [RNAi]; UAS-mitoGFP, D42-Gal4/UAS-marf[RNAi]	0.66 ± 0.04, n = 9	5.26 ± 0.70, n = 9	35.50 ± 1.9, n = 10	56.00 ± 4.51, n = 9

D42-Gal4 is a motor neuron driver. Values represent mean ± s.e.m.

Table J, related to S11 Fig: Electrophysiological properties for motor neuron MCI loss (or rescue) conditions.

Recording Condition	Genotype	Electrophysiological Data
0.15 mM Ca^{2+}	*D42-Gal4/+*	EPSP: 17.61 ± 4.16 mV, n = 10
	UAS-NDUFS7 [RNAi]/+; D42-Gal4/+	EPSP: 9.52 ± 2.01 mV, n = 11
Failure analysis:<br>0.1 mM Ca^{2+}<br><br>Data: % failures<br>n = number of muscles	*D42-Gal4/+*	18.08% ± 7.59, n = 13
	UAS-Sod2/+;D42-Gal4/+	27.00% ± 8.49, n = 8
	NDUFS7[RNAi]/+; D42-Gal4/+	1.87% ± 0.44, n = 8
	NDUFS7[RNAi]/+; D42-Gal4/+ with NACA	60.22% ± 15.16, n = 9
	NDUFS7 [RNAi]/UAS-Sod2; D42-Gal4/+	20.13% ± 8.18, n = 15
Paired pulse ratios (EPSP2/EPSP1)<br>0.4 mM Ca^{2+}	*D42-Gal4/+*	0.88 ± 0.06, n = 8
	NDUFS7 [RNAi]/+; D42-Gal4/+	0.82 ± 0.01, n = 10
Paired pulse ratios (EPSP2/EPSP1)<br>1.5 mM Ca^{2+}	*D42-Gal4/+*	0.88 ± 0.02, n = 12
	NDUFS7 [RNAi]/+; D42-Gal4/+	0.84 ± 0.01, n = 11

D42-Gal4 is a motor neuron driver. Values represent mean ± s.e.m.

Table K, related to Fig 4: BRP intensity, BRP density, electrophysiological parameters, and the number of mitochondrial clusters for genetic combinations shown in Fig 4.

**Genotype**	**BRP Intensity in AU (μm^2 area of bouton)**	**BRP density (per μm^2 area of bouton)**
UAS-mitoGFP, D42-Gal4/+	100.0 ± 7.83, n = 40	2.28 ± 0.06, n = 29
UAS-Sod2/+;<br>*UAS-mitoGFP, D42-Gal4/+*	84.29 ± 5.50, n = 38	2.29 ± 0.09, n = 30
UAS-NDUFS7 [RNAi]/+;<br>*UAS-mitoGFP, D42-Gal4/+*	195.2 ± 22.90, n = 42	1.78 ± 0.09, n = 30
NDUFS7 [RNAi]/+; UAS-mitoGFP, D42-Gal4/+<br>with NACA	110.8 ± 13.72, n = 41	2.22 ± 0.06, n = 30
NDUFS7 [RNAi]/UAS-Sod2;<br>*UAS-mitoGFP, D42-Gal4/+*	98.29 ± 7.96, n = 40	2.24 ± 0.10, n = 30
**Genotype**	**mEPSP amp (mV), mEPSP freq (Hz) EPSP (mV), and QC**	**# mito clusters (per μm^2 area of bouton)**
UAS-mitoGFP, D42-Gal4/+	mEPSP amp: 0.98 ± 0.04, n = 8,<br>mEPSP freq: 2.51 ± 0.12, n = 8<br>EPSP amp: 43.29 ± 1.75, n = 8<br>QC: 44.21 ± 0.72, n = 8	12.42 ± 1.03, n = 38
UAS-Sod2/+;<br>*UAS-mitoGFP, D42-Gal4/+*	mEPSP amp: 0.58 ± 0.023, n = 8,<br>mEPSP freq: 2.82 ± 0.56, n = 8<br>EPSP amp: 40.78 ± 0.50, n = 8<br>QC: 70.66 ± 3.02, n = 8	12.23 ± 0.78, n = 39
UAS-NDUFS7 RNAi]/+;<br>*UAS-mitoGFP, D42-Gal4/+*	mEPSP amp: 0.75 ± 0.03, n = 7,<br>mEPSP freq: 4.92 ± 0.81, n = 7<br>EPSP amp: 43.67 ± 1.55, n = 7<br>QC: 58.31 ± 3.92, n = 7	4.84 ± 0.44, n = 38
UAS-NDUFS7[RNAi]/UAS-Sod2;<br>*UAS-mitoGFP, D42-Gal4/+*	mEPSP amp: 0.65 ± 0.02, n = 14,<br>mEPSP freq: 3.18 ± 0.32, n = 14<br>EPSP amp: 37.62 ± 1.28, n = 14<br>QC: 58.06 ± 2.76, n = 14	5.48 ± 0.48, n = 41

D42-Gal4 is a motor neuron driver. Values represent mean ± s.e.m.

Table L, related to S12 Fig: Analysis of BRP intensity for various rotenone applications.

Genotype	BRP Intensity in AU (μm^2 area of a bouton)
w^{1118} + DMSO, nerve severed, 1 hour	100.0 ± 5.73, n = 75
w^{1118} + DMSO, nerve intact, 1 hour	76.18 ± 2.79, n = 74
w^{1118} + 500 µM ROT, nerve severed, 1 hour	100.0 ± 4.98, n = 68
w^{1118} + 500 µM ROT, nerve intact, 1 hour	105.3 ± 4.65, n = 77
w^{1118} + DMSO, nerve severed, 2 hours	100.0 ± 5.86, n = 80
w^{1118} + DMSO, nerve intact, 2 hours	97.54 ± 5.52, n = 80
w^{1118} + 500 µM ROT, nerve severed, 2 hours	100.0 ± 5.09, n = 75
w^{1118} + 500 µM ROT, nerve intact, 2 hours	113.1 ± 6.34, n = 81
w^{1118} + DMSO, nerve severed, 4 hours	100.0 ± 4.79, n = 78
w^{1118} + DMSO, nerve intact, 4 hours	105.9 ± 4.34, n = 70
w^{1118} + 500 µM ROT, nerve severed, 4 hours	100.0 ± 5.31, n = 60
w^{1118} + 500 µM ROT, nerve intact, 4 hours	95.41 ± 4.58, n = 78
w^{1118} + DMSO, nerve severed, 6 hours	100.0 ± 4.08, n = 71
w^{1118} + DMSO, nerve intact, 6 hours	92.90 ± 4.14, n = 70
w^{1118} + 500 µM ROT, nerve severed, 6 hours	100.0 ± 3.90, n = 90
w^{1118} + 500 µM ROT, nerve intact, 6 hours	134.8 ± 4.53, n = 80
w^{1118} + DMSO+8 hours feeding, intact larvae	100.0 ± 5.40, n = 80
w^{1118} + 25µM ROT+8 hours feeding, intact larvae	94.26 ± 3.84, n = 81
w^{1118} + DMSO+Embryo to 3rd instar	100.0 ± 3.88, n = 74
w^{1118} + 25µM ROT+Embryo to 3rd instar	121.9 ± 5.86, n = 64

ROT denotes rotenone; DMSO denotes dimethyl sulfoxide (carrier control). Values represent mean ± s.e.m.

Table M, related to S13 Fig: BRP intensity, electrophysiological parameters, and the number of mitochondrial clusters for genetic combinations shown.

**Genotype**	**BRP Intensity in AU (μm^2 area of a bouton)**	**# mito clusters (per μm^2 area of a bouton)**
UAS-mitoGFP, D42-Gal4/+	100.0 ± 1.91,n=26	6.53 ± 0.40, n=30
UAS-Cat/+; UAS-mitoGFP,D42-Gal4	87.80 ± 2.07, n=21	8.20 ± 0.45, n=30
UAS-Sod1/+; UAS-mitoGFP,D42-Gal4	81.85 ± 3.15, n=22	7.90 ± 0.68, n=30
NDUFS7[RNAi]/+; UAS-mitoGFP, D42-Gal4/+	148.9 ± 9.12, n=24	2.76 ± 0.30, n=30
NDUFS7[RNAi]/UAS-Cat; UAS-mitoGFP, D42-Gal4/+	128.0 ± 6.42, n=25	3.76 ± 0.38, n=30
NDUFS7 [RNAi]/UAS-Sod1; UAS-mitoGFP, D42-Gal4/+	152.9 ± 6.62, n=26	3.53 ± 0.33, n=30
**Genotype**	**mEPSP, EPSP (mV)**	**Quantal content**
UAS-mitoGFP, D42-Gal4/+	mEPSP amplitude: 0.76 ± 0.05, n=7, EPSP amplitude: 39.60 ± 1.07, n=7, mEPSP frequency: 2.74 ± 0.40 Hz, n=7	53.61 ± 4.48, n=7
UAS-Cat/+; UAS-mitoGFP, D42-Gal4/+	mEPSP amplitude: 0.59 ± 0.01, n=9, EPSP amplitude: 44.15 ± 1.15, n=9, mEPSP frequency: 2.76 ± 0.62 Hz, n=9	74.38 ± 2.65, n=9
UAS-Sod1/+; UAS-mitoGFP, D42-Gal4/+	mEPSP amplitude: 0.62 ± 0.02, n=11, EPSP amplitude: 43.27 ± 1.51, n=11, mEPSP frequency: 1.60 ± 0.26 Hz, n=11	70.34 ± 2.87, n=11
NDUFS7 [RNAi]/+; UAS-mitoGFP, D42-Gal4/+	mEPSP amplitude: 0.60 ± 0.028, n=7, EPSP amplitude: 42.18 ± 0.61, n=7, mEPSP frequency: 6.56 ± 0.87 Hz, n=7	70.43 ± 3.32, n=7
NDUFS7 [RNAi]/UAS-Cat; UAS-mitoGFP, D42-Gal4/+	mEPSP amplitude: 0.60 ± 0.023, n=12, EPSP amplitude: 42.96 ± 1.16, n=12, mEPSP frequency: 4.07 ± 0.67 Hz, n=12	72.33 ± 2.71, n=12
NDUFS7 [RNAi]/UAS-Sod1; UAS-mitoGFP,D42-Gal4/+	mEPSP amplitude: 0.54 ± 0.03, n=9, EPSP amplitude: 38.11 ± 0.69, n=9, mEPSP frequency: 2.65 ± 0.57 Hz, n=9	71.75 ± 4.27, n=9

D42-Gal4 is a motor neuron driver. Values represent mean ± s.e.m.

Table N, related to Fig 5: Electrophysiological properties for various genotypes: mEPSP amplitude, EPSP amplitude, mEPSP frequency, and QC.

**Genotype**	**mEPSP amplitude (mV)**	**EPSP amplitude (mV)**	**mEPSP frequency (Hz)**	**Quantal Content (QC)**
elaV-Gal4(C155/+)	0.63 ± 0.01, n = 7	38.22 ± 1.50, n = 7	1.90 ± 0.38, n = 7	60.65 ± 2.62, n = 7
elaV(C155)-Gal4/+ with XestC (20µM) & Dantrolene (10µM)	0.72 ± 0.05, n = 9	38.21 ± 1.29, n = 9	2.81 ± 0.58, n = 9	54.57 ± 3.44, n = 9
elaV(C155)-Gal4/+; UAS-NDUFS7 [RNAi]/+	0.79 ± 0.03, n = 10	39.96 ± 1.44, n = 10	3.83 ± 0.40, n = 10	51.45 ± 3.28, n = 10
elaV(C155)-Gal4/+; UAS-NDUFS7 [RNAi]/+ with XestC (20µM) & Dantrolene (10µM)	0.60 ± 0.04, n = 8	35.97 ± 1.05, n = 8	3.63 ± 0.58, n = 8	61.19 ± 5.29, n = 8
elaV(C155)-Gal4/+; UAS-CalX[RNAi]/+	0.80 ± 0.03, n = 8	35.45 ± 1.26, n = 8	1.61 ± 0.18, n = 8	44.42 ± 2.59, n = 8
elaV(C155)-Gal4/+; UAS-NDUFS7 [RNAi]/+; UAS-CalX[RNAi]/+	0.75 ± 0.03, n = 10	34.04 ± 1.72, n = 10	2.68 ± 0.40, n = 10	46.10 ± 3.10, n = 10
elaV(C155)-Gal4/+; UAS-IP$_3$-Sponge.m30/+ + DMSO	0.64 ± 0.04, n = 8	33.06 ± 2.84, n = 8	2.61 ± 0.51, n = 8	53.31 ± 6.23, n = 8
elaV(C155)-Gal4/+; UAS-IP$_3$-Sponge.m30/+ with XestC (20µM) & Dantrolene (10µM)	0.79 ± 0.03, n = 8	38.37 ± 0.96, n = 8	2.13 ± 0.36, n = 8	48.84 ± 2.96, n = 8
elaV(C155)-Gal4/+; UAS-NDUFS7 [RNAi]/+; IP3-Sponge.m30/+	0.67 ± 0.02, n = 10	35.55 ± 1.53, n = 10	3.70 ± 0.33, n = 10	53.60 ± 3.17, n = 10
elaV(C155)-Gal4/+; UAS-NDUFS7 [RNAi]/+; UAS-IP$_3$-Sponge.m30/+ with Dantrolene (10µM)	0.58 ± 0.02, n = 10	33.07 ± 1.09, n = 10	3.99 ± 0.50, n = 10	57.65 ± 3.22, n = 10
elaV(C155)-Gal4/+; UAS-mcu[RNAi]/+	0.77 ± 0.04, n = 9	34.87 ± 1.24, n = 9	3.39 ± 0.33, n = 9	46.49 ± 3.16, n = 9
elaV(C155)-Gal4/+; UAS-mcu[RNAi]/+ with XestC (20µM) & Dantrolene (10µM)	0.70 ± 0.08, n = 9	38.58 ± 1.25, n = 9	2.44± 0.21, n = 9	59.15 ± 5.97, n = 9

elaV(C155)-Gal4/+; UAS-NDUFS7 [RNAi]/UAS-mcu[RNAi]	0.70 ± 0.05, n = 9	32.26 ± 1.12, n = 10	3.83 ± 0.46, n = 9	48.87 ± 3.84, n = 9
elaV(C155)-Gal4/+; UAS-NDUFS7 RNAi]/UAS-mcu[RNAi] with XestC (20μM) & Dantrolene (10μM)	0.55 ± 0.04, n = 9	28.68 ± 1.63, n = 11	3.18 ± 0.40, n = 9	55.14 ± 7.01, n = 9
elaV(C155)-Gal4/+; UAS-NDUFS7 [RNAi]/ UAS-mcu[RNAi]/IP$_3$-Sponge.m30 with Dantrolene (10μM)	0.56 ± 0.05, n = 8	24.96 ± 2.91, n = 8	3.24 ± 0.42, n = 8	46.83 ± 6.20, n = 8
elaV(C155)-Gal4/+ + DMSO and wash	0.68 ± 0.01, n = 12	40.92 ± 2.38, n = 12	2.56 ± 0.35, n = 12	60.68 ± 4.04, n = 12
elaV(C155)-Gal4/+ + BAPTA-AM (20μM) and wash	0.66 ± 0.02, n = 13	40.38 ± 1.68, n = 13	2.21 ± 0.14, n = 13	61.94 ± 3.92, n = 13
elaV(C155)-Gal4/+; UAS-NDUFS7 [RNAi]/+ + DMSO and wash	0.59 ± 0.02, n = 14	43.39 ± 1.86, n = 14	3.28 ± 0.24, n = 14	74.81 ± 4.42, n = 14
elaV(C155)-Gal4/+; UAS-NDUFS7 [RNAi]/+ + BAPTA-AM (20μM) and wash	0.69 ± 0.06, n = 11	31.65 ± 2.92, n = 11	2.63 ± 0.43, n = 11	49.79 ± 6.74, n = 11

elaV(C155)-Gal4 is a pan-neuronal driver. Values represent mean ± s.e.m.

Table O, related to S14 Fig: Active zone marker intensity for genotypes in S14 Fig.

**Genotype**	**BRP Intensity in AU (μm² area of a bouton)**
elaV(C155)-Gal4/+ with DMSO	100.0 ± 6.96, n = 67
elaV(C155)-Gal4/+ with Xesto + Dant	100.1 ± 6.22, n = 86
elaV(C155)-Gal4/+; NDUFS7 [RNAi]/+ with DMSO	170.4 ± 14.26, n = 100
elaV(C155)-Gal4/+; NDUFS7 [RNAi]/+ with Xesto + Dant	142.7 ± 7.03, n = 100
elaV(C155)-Gal4/+; UAS-IP$_3$-sponge.m30/+	111.8 ± 6.02, n = 88
elaV(C155)-Gal4/+; mcu[RNAi]/+	100.3 ± 4.28, n = 111
elaV(C155)-Gal4/+; UAS-mcu[RNAi]/NDUFS7 [RNAi] with Xesto + Dant	64.78 ± 4.59, n = 83
elaV(C155)/+; UAS-mcu[RNAi]/NDUFS7[RNAi]/+; *UAS-IP$_3$-sponge.m30/*+ with Dant	85.73 ± 3.70, n = 100

elaV(C155)-Gal4 is a pan-neuronal driver. Xesto denotes XestosponginC. Dant denotes Dantrolene. Values represent mean ± s.e.m.

Table P, related to Fig 6: Electrophysiological properties and active zone marker intensity measurements for conditions depicted in Fig 6.

**Genotype**	**mEPSP amplitude (mV)**	**mEPSP frequency (Hz)**	**EPSP amplitude (mV)**	**Quantal content (QC)**	**BRP Intensity in AU (μm^2 area of a bouton)**
elaV(C155)-Gal4/+ +DMSO	0.60 ± 0.02, n = 10	4.34 ± 0.74, n = 10	41.52 ± 0.99, n = 10	68.70 ± 2.29, n = 10	100.0 ± 6.79, n = 69
elaV(C155)-Gal4/+ +LDA	0.69 ± 0.04, n = 9	4.93 ± 0.54, n = 9	41.22 ± 1.29, n = 10	60.59 ± 3.38, n = 9	193.1 ± 10.41, n = 94
elaV(C155)-Gal4/+ UAS-NDUFS7 [RNAi]/+ +DMSO	0.61 ± 0.03, n = 9	2.79 ± 0.27, n = 9	40.46 ± 0.82, n = 9	66.97 ± 3.72, n = 9	100.0 ± 4.65, n = 75
elaV(C155)-Gal4/+ UAS-NDUFS7 [RNAi]/+ +LDA	0.58 ± 0.02, n = 11	3.15± 0.25, n = 11	35.71 ± 0.56, n = 11	62.25 ± 2.95, n = 11	81.05 ± 5.37, n = 71
elaV(C155)-Gal4/+ +2-Deoxy-D-glucose	0.96 ± 0.04, n = 9	1.94 ± 0.22, n = 9	41.09 ± 1.66, n = 9	42.70 ± 1.52, n = 9	100.0 ± 4.71, n = 74
elaV(C155)-Gal4/+ UAS-NDUFS7 [RNAi]/+ +2-Deoxy-D-glucose	0.66 ± 0.07, n = 8	3.74 ± 1.02, n = 8	32.27 ± 1.72, n = 8	52.65 ± 5.82, n = 8	78.69 ± 3.80, n = 84

elaV(C155)-Gal4 is a pan-neuronal driver. LDA denotes lonidamine. Values represent mean ± s.e.m.

Table Q, related to S15 Fig: Electrophysiological parameters for conditions depicted in S15 Fig.

Genotype	mEPSP amplitude (mV)	mEPSP frequency (Hz)	EPSP amplitude (mV)	Quantal Content (QC)
elaV(C155)-Gal4/+	0.70 ± 0.04, n = 10	2.72 ± 0.52, n = 10	42.40 ± 1.19, n = 10	62.46 ± 3.71, n = 10
elaV(C155)-Gal4/+; UAS-NDUFS7 [RNAi]/+	0.97 ± 0.07, n = 10	2.92 ± 0.46, n = 10	42.11 ± 1.07, n = 11	47.33 ± 5.26, n = 10
elaV(C155)-Gal4/+; UAS-hex A[RNAi]/+	0.62 ± 0.02, n = 10	1.64 ± 0.17, n = 10	32.70 ± 1.75, n = 11	54.26 ± 3.85, n = 10
elaV(C155)-Gal4/+; UAS-hex C[RNAi]/+	0.67 ± 0.03, n = 10	1.84 ± 0.21, n = 10	44.69 ± 1.36, n = 10	67.60 ± 4.48, n = 10
elaV(C155)-Gal4/+; UAS-hex C[RNAi]/+; UAS-hex A[RNAi]/+	0.90 ± 0.04, n = 11	1.23 ± 0.09, n = 11	36.94 ± 3.09, n = 13	42.02 ± 4.65, n = 11
elaV(C155)-Gal4/+; UAS-idh[RNAi]/+	0.71 ± 0.03, n = 10	1.79 ± 0.27, n = 10	48.58 ± 1.60, n = 10	69.15 ± 3.67, n = 10
elaV(C155)Gal4/+; UAS-Cit.(Si)-Syn[RNAi]/+	0.87 ± 0.09, n = 9	1.67 ± 0.14, n = 9	44.61 ± 1.98, n = 9	53.81 ± 3.20, n = 9
elaV(C155)-Gal4/+; UAS-Scsα[RNAi]/+	0.83 ± 0.04, n = 8	1.82 ± 0.37, n = 8	42.33 ± 2.81, n = 9	50.19 ± 3.47, n = 8
elaV(C155)-Gal4/+; UAS-NDUFS7 [RNAi]/+; UAS-hex A[RNAi]/+	0.62 ± 0.02, n = 12	2.99 ± 0.33, n = 12	31.10 ± 0.81, n = 13	51.33 ± 2.43, n = 12
elaV(C155)-Gal4/+; UAS-NDUFS7 [RNAi]/+; UAS-hex C[RNAi]/+	0.74 ± 0.04, n = 12	3.39 ± 0.33, n = 12	37.93 ± 1.37, n = 12	53.38 ± 3.66, n = 12
elaV(C155)-Gal4/+; UAS-NDUFS7 RNAi]/UAS-hex C[RNAi]; UAS-hex A[RNAi]/+	0.99 ± 0.05, n = 12	2.60 ± 0.48, n = 12	27.70 ± 1.86, n = 13	28.48 ± 2.10, n = 12
elaV(C155)-Gal4/+; UAS-NDUFS7 [RNAi]/+; UAS-idh[RNAi]/+	0.62 ± 0.01, n = 12	3.40 ± 0.49, n = 12	41.34 ± 1.72, n = 12	66.30 ± 2.58, n = 12
elaV(C155)-Gal4/+; UAS-NDUFS7 [RNAi]/+; UAS-Cit.(Si)-Syn[RNAi]/+	0.68 ± 0.06, n = 14	3.95 ± 0.72, n = 14	32.73 ± 1.87, n = 14	51.82 ± 4.85, n = 14
elaV(C155)-Gal4/+; UAS-NDUFS7 [RNAi]/+; UAS-Scsα[RNAi]/+	0.74 ± 0.04, n = 8	3.09 ± 0.66, n = 8	29.90 ± 1.20, n = 8	41.45 ± 3.17, n = 8

elaV(C155)-Gal4 is a pan-neuronal driver. Values represent mean ± s.e.m.

Table R, related to S16 Fig: Active zone intensity data for genotypes in S 16 Fig.

**Genotype**	**BRP Intensity in AU (µm^2 area of a bouton)**
elaV(C155)-Gal4/+	100.0 ± 6.74, n = 68
elaV(C155)-Gal4/+; UAS-NDUFS7 [RNAi]/+	218.3 ± 10.89, n = 96
elaV(C155)-Gal4/+; UAS-hex A[RNAi]/+	209.6 ± 11.43, n = 69
elaV(C155)-Gal4/+; UAS-hex C[RNAi]/+	93.20 ± 7.22, n = 68
elaV(C155)-Gal4/+; UAS-hex C[RNAi]/+; UAS-hex A[RNAi]/+	123.3 ± 5.68, n = 84
elaV(C155)-Gal4/+; UAS-idh[RNAi]/+	223.0 ± 14.31, n = 98
elaV(C155)-Gal4/+; UAS-Cit.(Si)-Syn[RNAi]/+	187.1 ± 12.40, n = 79
elaV(C155)-Gal4/+; UAS-Scsα[RNAi]/+	155.8 ± 11.47, n = 86
elaV(C155)-Gal4/+; UAS-NDUFS7 [RNAi]/+; UAS-hex A[RNAi]/+	54.37 ± 4.41, n = 63
elaV(C155)-Gal4/+; UAS-NDUFS7 [RNAi]/UAS-hex C[RNAi]	121.7 ± 11.47, n = 68
elaV(C155)-Gal4/+; UAS-NDUFS7 [RNAi]/UAS-hex C[RNAi]/+; UAS-hex A[RNAi]/+	120.2 ± 6.61, n = 84
elaV(C155)-Gal4/+; UAS-NDUFS7 RNAi]/+; UAS-idh[RNAi]/+	81.01 ± 4.54, n = 87
elaV(C155)-Gal4/+; UAS-NDUFS7 [RNAi]/+; UAS-Cit.(Si)-Syn[RNAi]/+	138.2 ± 8.38, n = 90
elaV(C155)-Gal4/+; UAS-NDUFS7 [RNAi]/+; UAS-Scsα[RNAi]/+	73.09 ± 5.49, n = 75

elaV(C155)-Gal4 is a pan-neuronal driver. Values represent mean ± s.e.m.

Table S, related to Fig 7: NMJ developmental parameters for genotypes shown in Fig 7.

Genotype	# boutons	Muscle area (μm^2)	# of branches	Relative Dlg area (μm^2)	Bouton area (μm^2)
BG57-Gal4/+	94.38 ± 5.09, n = 8	59050 ± 2845, n = 8	12.00 ± 1.01, n = 8	8.39 ± 0.70, n = 30	19.20 ± 1.15, n = 30
UAS-Sod2/+; BG57-Gal4/+	83.38 ± 4.76, n = 8	56340 ± 1447, n = 8	8.12 ± 0.78, n = 8	6.25 ± 0.38, n = 30	18.11 ± 0.84, n = 30
UAS-Sod1/+; BG57-Gal4/+	83.89 ± 5.39, n = 9	54490 ± 2001, n = 9	7.55 ± 0.70, n = 9	7.05 ± 0.69, n = 29	22.44 ± 1.30, n = 29
UAS-Cat/+; BG57-Gal4/+	79.50 ± 5.80, n = 8	56190 ± 2297, n = 8	8.50 ± 0.50, n = 8	9.60 ± 0.77, n = 30	26.26 ± 1.16, n = 30
UAS-NDUFS7 RNAi/+; BG57-Gal4/+	61.88 ± 5.57, n = 8	34250 ± 2546, n = 8	7.00 ± 0.70, n = 8	2.36 ± 0.23, n = 30	10.40 ± 0.59, n = 30
UAS-NDUFS7 [RNAi]/+; BG57-Gal4/+ +0.5mM NACA	92.38 ± 5.09, n = 8	35990 ± 1377, n = 8	8.62 ± 0.56, n = 8	5.63 ± 0.42, n = 30	15.27 ± 0.93, n = 30
UAS-NDUFS7 RNAi]/ UAS-Sod2; BG57-Gal4/+	87.88 ± 5.83, n = 8	35850 ± 1967, n = 8	9.00 ± 0.68, n = 8	7.55 ± 0.43, n = 34	15.59 ± 0.75, n = 34
UAS-NDUFS7 [RNAi]/ UAS-Sod1; BG57-Gal4/+	58.75 ± 4.17, n = 8	31620 ± 2084, n = 8	5.75 ± 0.31, n = 8	3.53 ± 0.50, n = 30	14.36 ± 0.93, n = 30
UAS-NDUFS7 [RNAi]/ UAS-Cat; BG57-Gal4/+	58.25 ± 3.80, n = 8	31180 ± 1069, n = 8	6.37 ± 0.26, n = 8	3.91 ± 0.35, n = 30	14.52 ± 0.73, n = 30
elaV(C155)-Gal4/+	89.75 ± 5.00, n = 8	70710 ± 4063, n = 9	7.00 ± 0.37, n = 8	5.05 ± 0.37, n = 22	10.82 ± 0.78, n = 22
elaV(C155)-Gal4/+; UAS-NDUFS7 [RNAi]/+	113.8 ± 7.81, n = 8	74980 ± 2586, n = 8	9.25 ± 0.83, n = 8	4.23 ± 0.45 n = 25	10.10 ± 0.62, n = 25
D42-Gal4/+	98.22 ± 9.30, n = 9	70850 ± 2785, n = 9	7.88 ± 1.04, n = 9	5.51 ± 0.40, n = 25	8.59 ± 0.38, n = 25
UAS-NDUFS7 [RNAi]/+; D42-Gal4/+	121.4 ± 4.58, n = 8	68390 ± 3436, n = 8	9.87 ± 0.93, n = 8	4.92 ± 0.45, n = 25	7.95 ± 0.53, n = 25

BG57-Gal4 is a muscle driver. *elaV(C155)-Gal4* is a pan-neuronal driver. *D42-Gal4* is a motor neuron driver. Values represent mean ± s.e.m.

Table T, related to Fig 9: Electrophysiological parameters for genotypes and conditions in Fig 9.

Genotype	mEPSP amplitude (mV)	mEPSP frequency (Hz)	EPSP amplitude (mV)	Quantal Content (QC)
BG57-Gal4/+	0.77 ± 0.02, n = 8	4.54 ± 0.36, n = 8	37.33 ± 1.60, n = 8	48.63 ± 2.60, n = 8
NDUFS7 [RNAi]/+; BG57-Gal4/+	0.63 ± 0.05, n = 7	5.45 ± 0.76, n = 7	25.49 ± 1.31, n = 7	41.97 ± 4.12, n = 7
NDUFS7 [RNAi]/UAS-Cat; BG57-Gal4/+	0.79 ± 0.06, n = 8	9.27 ± 0.74, n = 8	25.47 ± 2.20, n = 8	33.07 ± 3.39, n = 8
NDUFS7 [RNAi]/ UAS-Sod1 BG57-Gal4/+	0.77 ± 0.07, n = 8	5.28 ± 0.73, n = 8	26.34 ± 1.82, n = 8	37.15 ± 5.05, n = 8
NDUFS7 [RNAi]/UAS-Sod2 BG57-Gal4/+	0.77 ± 0.07, n = 6	6.99 ± 1.21, n = 6	33.26 ± 1.08, n = 6	38.24 ± 6.79, n = 6
BG57-Gal4/+ +10% EtOH	0.77 ± 0.04, n = 9	4.79 ± 0.36, n = 9	37.77 ± 2.00, n = 9	50.17 ± 3.92, n = 9
NDUFS7 [RNAi]/+; BG57-Gal4/+ 10% EtOH	0.62 ± 0.05, n = 12	7.12 ± 1.30, n = 12	27.49 ± 1.20, n = 10	43.65 ± 4.06, n = 10
NDUFS7 RNAi]/+; BG57-Gal4/+ + 0.5mM Curcumin	0.69 ± 0.06, n = 9	6.07 ± 0.85, n = 9	29.74 ± 1.28, n = 9	46.00 ± 5.05, n = 9
NDUFS7 [RNAi]/+; BG57-Gal4/+ 0.5mM NACA	0.88 ± 0.037, n = 9	5.34 ± 0.88, n = 9	35.12 ± 1.23, n = 9	39.88 ± 1.37, n = 9
ND-30 mutant (*ND-30 ^{epgy}/Df*)	0.50 ± 0.02, n = 10	6.74 ± 1.11, n = 10	20.27 ± 1.72, n = 10	41.22 ± 4.34, n = 10
ND-30 ^{epgy}/Df + 0.5 mM NACA	0.71 ± 0.06, n = 8	6.50 ± 1.17, n = 8	30.32 ± 1.36, n = 8	44.59 ± 4.20, n = 8
Sod2 muscle rescue *UAS-Sod2/+; ND-30 ^{epgy}/Df, BG57-Gal4*	0.56 ± 0.04, n = 8	5.07 ± 0.34, n = 8	36.19 ± 1.08, n = 8	66.13 ± 4.42, n = 8
BG57-Gal4/+ + 0.5% DMSO	0.92 ± 0.03, n = 9	4.43 ± 0.44, n = 9	40.09 ± 1.95, n = 9	43.04 ± 0.79, n = 9
BG57-Gal4/+ + 50µM rotenone	0.94 ± 0.08, n = 9	4.36 ± 0.71, n = 9	31.96 ± 1.43, n = 9	35.50 ± 3.08, n = 9
UAS-Cat/+; BG57-Gal4/+ + 50µM rotenone	0.72 ± 0.03, n = 11	5.10 ± 0.52, n = 11	31.58 ± 2.40, n = 11	44.34 ± 3.67, n = 11
UAS-Sod1/+; BG57-Gal4/+ + 50µM rotenone	0.82 ± 0.03, n = 8	4.53 ± 0.46, n = 8	30.07 ± 1.86, n = 8	36.63 ± 2.30, n = 8
UAS-Sod2/+; BG57-Gal4/+ + 50µM rotenone	0.71 ± 0.05, n = 7	5.29 ± 0.49, n = 7	36.54 ± 1.55, n = 8	54.15 ± 4.60, n = 7

BG57-Gal4 is a muscle driver. Values represent mean ± s.e.m.

Table U, related to Fig 9: Crawling behavior of various MCI loss or rescue combinations.

Genotype	Distance crawled in cm
BG57-Gal4/+	2.55 ± 0.09, n = 12
UAS-Sod2/+; BG57-Gal4/+	2.72 ± 0.18, n = 15
UAS-ND-30[RNAi]/BG57-Gal4	1.83 ± 0.12, n = 15
UAS-NDUFS7 [RNAi]/+; BG57-Gal4/+	0.79 ± 0.06, n = 10
UAS-NDUFS7 [RNAi]/UAS-Sod2; BG57-Gal4/BG57-Gal4	1.49 ± 0.15, n = 12
UAS-NDUFS7 [RNAi]/+; BG57Gal4/+ +NACA	1.60 ± 0.13, n = 8
elaV(C155)-Gal4/+	2.47 ± 0.14, n = 10
elaV(C155)-Gal4/+ ;NDUFS7 [RNAi]/+	2.51 ± 0.14, n = 10
ND-30^{epgy}/Df	1.34 ± 0.15, n = 9
UAS-Sod2/+;ND-30^{epgy}/Df,BG57-Gal4	1.85 ± 0.15, n = 9

BG57-Gal4 is a muscle driver. *elaV(C155)-Gal4* is a pan neuronal driver. *Df* denotes the chromosomal deficiency *Df(3L)ED4288*, which uncovers the *ND-30* endogenous locus. Values represent mean ± s.e.m.

Table V, related to STAR Methods section: Reagents.

***Drosophila* gene name and Bloomington *Drosophila* stocks (stock number)**	**Antibodies (dilution), Drugs, and Fluorophores**
UAS-ND-30[RNAi]/CG12079 (BL 44535)	Mouse α-BRP (DSHB-1:30),<br>Mouse α-DLG (DSHB-1:50)
UAS-NDUFS7 [RNAi]/CG2014 (BL 62381)	Mouse α-Alpha Spectrin (DSHB-1:20)
UAS-marf [RNAi] (BL 67158),<br>*UAS-drp1[RNAi]* (BL 51483)	Mouse α-CSP (DSHB-1:50),<br>Mouse α-Synapsin (DSHB-1:30)
UAS-mitoGFP (BL 8442),<br>*UAS-mitoGFP, D42* (BL 42737)	Mouse α-GluRIIA (DSHB-1:50)
UAS-Sod1 (BL 33605),<br>*UAS-Sod2* (BL 24494),<br>*UAS-Catalase* (BL 24621)	Mouse α-22C10 (DSHB-1:50)
Df(3L)ED4288 (BL 8057),<br>$ND\text{-}30^{epgy} = ND\text{-}30^{EY03664}$ (BL 16569)	Polyclonal rabbit α-GFP (Abcam: 1:250)<br>Polyclonal rabbit α-GluRIII<br>(Dr. Aaron DiAntonio lab-1:100)<br>Polyclonal rabbit α-DLG (1:700)
elaV(C155-Gal4) (BL 458),<br>*D42-Gal4* (BL 8816)	Dantrolene (Tocris: 10 µM),<br>Xestospongin C (Abcam: 20 µM)<br>Rotenone (Sigma aldrich: 50 µM),<br>Curcumin (Sigma aldrich: 0.5 mM)
BG57-Gal4 (BL 32556)	NACA (Sigma Aldrich: 0.5mM), MitoSOX™<br>Red (Molecular Probes,<br>Thermo Fisher Scientific, 1:200),<br>BAPTA-AM (Sigma Aldrich)<br>Lonidamine (LDA: 150 µM Sigma Aldrich)<br>2-Deoxy-D-glucose (Sigma Aldrich)
UAS-CalX [RNAi] (BL 28306),<br>*UAS-mcu[RNAi]* (BL 42580, BL 67857),<br>*UAS-IP3-Sponge.m30* (Koganezawa lab)<br>*UAS-hexokinase A[RNAi]* (BL 35155)<br>*UAS-hexokinase C[RNAi]* (BL 57404)<br>*UAS-idh[RNAi]* (BL 41708)<br>*UAS-Cit.(Si)-Syn[RNAi]* (BL 36740)<br>*UAS-Scsα[RNAi]* (BL 51807)<br>*UAS-Sod2[RNAi]* (BL 24489)	Alexa α-HRP 488 (1:800), Rhodamine α-HRP (1:200),<br>Alexa α-HRP 647 (1:200),<br>Mouse and Rabbit Alexa Fluor 488 or 568 (1:400)
