# Supplementary material for: Mitochondrial Complex I and ROS control neuromuscular function through opposing pre- and postsynaptic mechanisms
Source: PLoS Biol. 2025 Sep 22;23(9):e3003388. doi: 10.1371/journal.pbio.3003388 (PMC12478897; doi:10.1371/journal.pbio.3003388)
Supplement: Attachment — Submitted filename: Response to Reviewers PBIOLOGY.docx [file pbio.3003388.s024.docx]

**RESPONSE TO REVIEWS**

We appreciate the time and consideration that all three reviewers put into evaluating our initial submission. We also appreciate the opportunity to revise our manuscript for *PLOS Biology*. In response to the initial round of review, we have done the following:

1. From Reviewer #1, there were concerns about study design. We provided a response to the critiques (see below). We shared many of the same considerations as Reviewer #1 before we embarked on our study, and we tried to incorporate that thinking into our design. We highlighted parts of the dataset and revision responsive to specific concerns. Additionally, we edited text in relevant sections.

2. For Reviewer #2’s comments, there were a few experimental suggestions centered on ROS scavengers, as well as requests for some revisions, mostly related to text and referencing.

- We made the requested revisions.
- We tested ROS scavengers SOD1 and Catalase for possible effects in suppressing neuronal phenotypes. As was the case with muscle, neither SOD1 nor Catalase suppressed neuronal MCI phenotypes.

3. For Reviewer #3’s comments, we addressed important concerns mainly through new experiments.

- We demonstrated an on-target depletion of both *Drosophila* NDUFS7 homologs using the *UAS-ND-20L[RNAi]* line, including *ND-20*. We believe that this solves the conundrum articulated by Reviewer #3 about the tissue specificity of MCI subunits (see below for specifics).
- We edited the relevant text and figures to reflect the new information. The RNAi-mediated knockdowns using the *ND-20L[RNAi]* line are now referred to as *NDUFS7[RNAi]*.
- We measured cellular metabolism in muscles after MCI knockdown using a Seahorse assay. It is significantly diminished.
- We demonstrated that genetic knockdown of *Sod2* yielded high levels of mitochondrial ROS.
- We added reagent citations where appropriate.
- We edited the text and figures to clarify several points.

***REVIEWS:***

***Reviewer #1: The authors report the consequences of suppressing the expression of one of the subunits of  Complex I, the first enzyme complex in the electron transport chain, on both mitochondrial, muscle and presynaptic biology in drosophila larvae.  On the whole the results appear robust but the logic of the manuscript is somewhat flawed and it makes interpretation difficult.  The works reads somewhat like an encyclopedic account of what happened for a particular perturbation without nailing down much in the way of mechanism.***

We appreciate Reviewer #1’s assessment. At a top level, we respectfully disagree in terms of the mechanistic contribution. We also disagree that the logic driving the manuscript was flawed. Our work began with the straightforward observation that Mitochondrial Complex I dysfunction induced synaptic phenotypes. We published that finding (Mallik and Frank, 2022). For our current investigation, we wanted to follow up on that initial observation. We found cellular and molecular details to explain those synaptic phenotypes. In our opinion, the following details constitute a mechanistic advance:

- Mitochondrial ROS accumulation drove synaptic phenotypes.
- ROS played either an adaptive or a destructive role, depending on whether the mitochondrial disruption was targeted to neurons or muscles. This was a mechanistic advance.
- ROS triggered specific, visible changes at pre- and postsynaptic sites, depending on its accumulation in neurons or muscle. Those changes matched corresponding electrophysiological experiments in the same genotypes. On the level of synapses, this was a mechanistic advance.
- The data were complementary. They included physiological, cytological, molecular, pharmacological, and genetic. This multi-pronged approach allowed us to produce (and to reproduce) mechanistic conclusions in multiple ways.
- The approach allowed us to address mitochondrial pleiotropies in mechanistic ways. We did this not only by examining a single mitochondrial perturbation – but also by combining it with additional genetic, molecular, or pharmacological perturbations.
- Our major conclusions were not obvious beforehand. This fact represents a conceptual advance.

***The major difficulties in lending interpretation to the findings stem from the following: There are several consequences one would expect for a mitochondrial perturbation: 1) a bioenergetic consequence, i.e. the sequalae of insufficient production of ATP, 2) related to the above but potentially different, the loss of cataplerotic or  increase in anaplerotic  metabolites that normally are exported or imported  from/into mitochondria; 3) improper Ca handling due to the loss of a major potential Ca buffer, i.e. the mitochondrial  transient uptake of Ca  via MCU; 4) the consequences of deleterious handling of oxygen within the electron transport chain resulting in an over-abundance of reactive oxygen.***

We agree with the idea that pleiotropic effects result from perturbing mitochondria. But if we are reading this comment correctly, the argument seems to be that it would be difficult to interpret anything at all from our data because mitochondrial perturbations are pleiotropic. We disagree with that part.

When MCI initially emerged from our synaptic function screen (Mallik and Frank, 2022), we also wondered about possible complications due to pleiotropies. That is why we ran tests limiting (or eliminating) other sources of ATP. It is also why we conducted experiments manipulating ROS, calcium signaling molecules, or MCU.

We appreciate the general concern of Reviewer #1 – mitochondrial pleiotropies are varied and complex. Given that backdrop, it was incumbent on us to examine several parameters. But by examining them piece by piece, we believe that we were able to make specific conclusions that were new.

***A priori one would not, from a pure bioenergetic point of view, expect that the stimulus paradigm used here would be a particularly stringent test of a bioenergetic compromise that one might anticipate from a mitochondrial perturbation. Perhaps more problematic is that the authors are using unrealistic concentration of the main consumable external carbon source, in this case 115 mM sucrose, perhaps 2 orders of magnitude too high. Unfortunately, it is very hard to understand what functional impact anything might mean when probing function under these metabolic conditions and with relatively sparsely demands (as in the case of Fig 1) or even during repetitive stimulation (Fig S6)- the latter though has additionally modified another parameter, external Ca.***

We appreciate this concern. This comment has different levels to it, and we can address them.

First, regarding 115 mM sucrose: That concentration of sugar of was not chosen arbitrarily. It dates to the 1990s, with experiments characterizing HL3 saline (HL – hemolymph-like) (Stewart *et al.*, 1994. J. Comp. Physiol. A. *175*, 179-191). The authors of Stewart *et al.* 1994 characterized many different saline conditions for NMJ electrophysiology. They endorsed HL3 because it maintained a healthy NMJ prep for long enough to conduct robust recordings, without the need for artificial cooling. HL3 has 115 mM sucrose, which helps to maintain the osmolarity of the preparation.

Today, *Drosophila* electrophysiologists use HL3 routinely. Importantly, 30+ years of research shows that synapse defects – both major and minor – are readily revealed at the *Drosophila* NMJ in HL3 saline with 115 mM sucrose. This includes experiments in low calcium and single stimulus events.

Second, regarding bioenergetics and synaptic transmission: Reviewer #1 could be right – even though HL3 is a well-established saline for electrophysiology, the neuronal tissues in the prep may still use the sucrose from the saline to cover for loss of MCI. But we considered that possibility in the original draft. Specifically, we considered the idea that the sucrose and trehalose in the saline could support glycolysis, thereby masking major defects that (otherwise) would have been caused by neuronal MCI dysfunction.

For the experiments in Figure 6, we removed all sucrose and trehalose from the saline and replaced it with 2-Deoxy-D-Glucose, which is a non-glycolytic sugar. This allowed us to maintain the osmolarity of the prep, while removing external sources of glycolysis. That revealed a partial role for energy/sugar contribution (Figure 6). This idea was buttressed by concurrent knockdown of MCI and glycolysis or TCA cycle genes (Revision Figure S15).

Third, as the reviewer notes, we ran a stringent electrophysiology test for Revision Figure S11 (original Figure S6). That was an important test. Our lab and others have observed that this repetitive stimulation paradigm in physiological calcium (10 Hz x 6000 pulses = 10 minutes; HL3 with 2 mM Ca^2+^) is around the maximum that a wild-type NMJ preparation can handle before starting to exhaust the recycling machinery and diminishing evoked potentials (e.g., see Frank et al., 2006). However, in our study with MCI knocked down in neurons, the preparation still maintained function, even under such a test.

***With respect to Ca handling the authors have made use of a few tools to interrupt contributions of ER stores, but in the absence of some functional measure of Ca in either the ER, the mitochondrial matrix or the cytoplasm (ideally all 3) , these are not easy to interpret.***

We tried to be parsimonious with our conclusions here, and we regret if anything we wrote was misleading. The interpretation of our experiments for this part of the paper is in the section header: “Neuronal MCI subunits stabilize synaptic strength in conjunction with intracellular calcium signaling proteins.” We believe that this interpretation is conservatively stated, given that we used five tools to impair steps of intracellular calcium handling in Figure 5.

The reviewer suggests a good experiment. The best way to do it would be to use genetically encoded calcium indicators and to target them to each separate cellular component to measure local changes in calcium concentration. In our opinion, it would be beyond the scope of this paper to do that. The scope of the current paper is how a synapse in general responds to mitochondrial dysfunction. For the revised Discussion in the portion that addresses intracellular calcium, we noted that these cellular compartments could be scrutinized to make more refined conclusions.

***The other major complexity arises from the fact that although ROS scavenging (or the lack thereof) has impact on presynaptic biology, it is very unclear if any of this is actually a local effect or one that arises in the cell body. As even the chemical scavenging perturbation is done over the time scale of development (actually not precisely stated but I am inferring this from the statement "grown in" in the methods). Thus, although conditions that lead to excess ROS production appear to drive interesting changes in presynaptic organization, whether this arose locally from axonal or presynaptic mitochondria or was a transcriptional response in the cell body is very unclear.***

***In this regard  it could simply be that the deleterious ROS impact in muscles is also confined to the cell body, but given that this is not spatially very separate from the endplate means that the consequences are different that in neurons but the reason may be more spatial than anything else.***

These are helpful comments in terms of what we could have done to communicate our ROS experiments better. We are grateful to have a chance to answer the concern and to revise some of our manuscript text. Again, there are multiple levels to Reviewer #1’s comment.

First, regarding the timing of ROS scavengers, the reviewer is correct. When we provided a chemical scavenger, we supplemented the fly food with it, and therefore, the exposure was over the course of development. We have clarified our Methods about this point.

Second, regarding the subcellular site of ROS, the reviewer’s main idea is right: one cannot pinpoint the subcellular site of action of ROS for most of these experiments. We did not want to overstate our findings; thus, we tried to avoid phrases like “presynaptic ROS” or “postsynaptic ROS” when describing our results. We searched our original manuscript, and we found three instances where we used those two phrases. We edited all three for the revision, and we have added comments in the Limitations and Future Directions section in the Discussion about not knowing the subcellular site of ROS.

We should note that our experiments *can* distinguish “neuronal ROS” vs. “muscle ROS” because of the tissue-specific manipulations to control where the mitochondrial perturbation (or scavenging event) was happening. Those manipulations do redound to impacts on presynaptic biology – and we believe that this fact is worth reporting, regardless of where the primary subcellular ROS site is.

Third, regarding muscle cell bodies (and ROS) potentially being physically closer to synaptic sites, this is a valid hypothesis, and we thank the reviewer for bringing it up. We have incorporated this idea into Discussion section as a way of comparing what happens in neurons vs. muscle.

Finally, we conducted a variation of the experiment the reviewer suggests. In the original manuscript, those data were housed in Figure S7, and they are now in Revised Figure S12. Acutely applying rotenone to the preparation (up to 4 hours) did not affect the presynaptic active zone protein levels. Application of rotenone to the preparation for 6 hours did induce an enhancement of active zone protein levels, but only if the motor nerve was intact. That does not pinpoint the relevant subcellular site of mitochondrial ROS downstream of rotenone. However, it does suggest that whatever signal is generated by ROS, it takes time and space in the neuron for that signal to trigger active zone material to traffic to presynaptic sites of release.

***At this stage this manuscript is not ready for publication without trying to attack these points in a logical fashion.  I am surprised the authors did not try to make better use of tools like rotenone, a bona fide Complex I inhibitor. Comparisons of the consequence of more acute pharmacological intervention compared to a chronic genetic one might lead to greater insight and interpretability of the results.***

Our dataset and conclusions are not based on a single reagent or approach. We mostly used genetic approaches because this was the appropriate way to test tissue-specific contributions of MCI.

To the reviewer’s point, we also made considerable use of rotenone. The example above from Figure S7 in the original manuscript was a case of applying rotenone for different amounts of time until presynaptic active zone changes became apparent. Additionally, in Mallik and Frank, 2022, we applied a high concentration of rotenone (500 µM) directly to the NMJ and found a small defect in neurotransmission after 30 minutes. Taking the two studies together, it seems that it takes the open prep several hours to respond to rotenone challenge and adjust active zone material.

Rotenone is a *bona fide* way to inhibit Complex I, but it is not a tissue-specific manipulation. A heavy reliance on rotenone might not be easy to interpret either. This is because one cannot know its primary tissue of action for any given phenotype. This is why we pursued the tissue-specific knockdowns of Complex I components in this study, in addition to using rotenone.

*****************************

***Reviewer #2 (Sean Sweeney): Mallik and Frank have produced and extensive and wide-ranging study of ROS, mitochondrial function, calcium regulation at the pre- and post-synaptic compartment. While manipulating mitochondrial complex I (MCI) in the presynaptic compartment, they identify changes in mitochondrial shape, networks and distribution. Changes in microtubule structures and increases in pre-synaptic active zones are also observed and these are rescued via expression of mitochondrially localised SOD2. With MCI manipulation there is no overt change to the synaptic output, but a change is revealed when the manipulation is rescued with SOD2 expression. In the presence of MCI knockdown, manipulations of ER Ca2+ (inhibition) reveal deficits in EPSP that are not present unless MCI is knocked down. When reading, I was then asking what ATP levels looked like, but the authors took a different but complementary route to check this: Glycolysis also appears to play an active role, in the absence of EPSP deficits when MCI is knocked down, also regulating active zone levels.***

We thank Reviewer #2 (Dr. Sweeney) for the positive assessment. Reviewer #3 shared similar thoughts about ATP. In response to Reviewer #3, we took a more direct route to address mitochondrial oxygen consumption, using a Seahorse assay and mitochondria collected from muscle tissue.

***The study then switches to muscles and the manipulation of MCI there. Synapse overgrowth, decrease in EPSP, loss of the PSD95 protein (dlg) and GluR puncta are observed, and critically some of these aspects can be rescued by SOD2 expression or NACA feeding, but not SOD1 (approximates to cytoplasmic ROS) or catalase.***

***There is much more, and a lot to digest in this paper, but I feel it is pretty comprehensive. I would have liked to see if the rescues with SOD1 or catalase also failed to rescue the pre-synaptic phenotypes, and if these attempts also rescued GluRIII loss. However, I feel the paper would stat to become unwieldy and the authors have made some very important novel points regarding the actions of ROS in the nervous system. The paper is very clearly written and easy to follow (tho the volume of critical data is large!), the images and graphs clear.***

Thank you, Dr. Sweeney. We also wish to avoid the paper becoming unwieldy. In the end, we were able to add the suggested experiments about SOD1 and catalase to the presynaptic part of the story. The answer is the same as before: in both neurons and muscle, only SOD2 or NACA seem to reverse the phenotypes due to ROS. SOD1 and catalase do not. These experiments help our understanding of the synaptic landscape, and we have included them with the revision.

***I have a couple of minor suggestions, typos:***

***Figure 1, L and M, the shape of the nuclei in M (I assume they are nuclei?) suggests this part of the prep is a bit squished, perhaps improve image M? Or is nuclear shape part of the phenotype? If so, please mention this in the text.***

The prep is not squished – rather, it is the case that muscle disorganization is a part of the phenotype that often emerged with muscle impairment of MCI. We include this disorganization description and the oblong-shaped nuclei in the Results text.

***Fig 4, is there an internal control for the brp staining for quantification?***

Yes, the Brp quantification was controlled by immunostaining all the preps for all the compared genotypes in the same tube. We distinguished genotypes of the final stained preps by how each was cut when dissected (e.g., head + tail; no head; no tail; no head + no tail; etc.). When preps were mounted, they were imaged using the same settings. Control levels of fluorescence intensity per unit area were normalized as 100 and the remaining genotypes were quantified by intensity, relevant to that.

***Line 700 Cystine should be Cysteine.***

Thank you, we have fixed this.

***Line 711, due to induction of brp??***

Thank you, what we intended to convey was that the EPSP levels were likely maintained due to the increased levels of active zone material (“induction”). We have rephrased this for the revision.

***Fig 5, increase the size of the green arrows and red crosses to make the figures work - they are the operative symbols and need to dominate a little more.***

We believe that the reviewer means the cartoon depicting intracellular calcium handling molecules. Given how the symbols are layered (in a now-flattened image), this is not a straightforward adjustment. However, given small edits to our text and legend, we hope that the figure is sufficiently clear.

***In the main text - please give a rationale for the switch between the OK371-GAL4 driver to the Elav-GAL4 driver. The switch needs to be explained.***

The switch from a motor neuron-specific driver (in this case it was *D42-Gal4*, not *OK371-Gal4*) to a pan-neuronal driver (*elaV-Gal4*) occurred between Figures 4 and 5. For the earlier figures, it made sense to confine manipulations to motor neurons, especially for imaging them. The switch to *elaV-Gal4* for later experiments was a practical decision for genetics – the number of *Drosophila* chromosomes carrying a manipulation was high from Figure 5 onward, so it was helpful to use *elaV(C155)-Gal4*, which is conveniently housed on the X chromosome. We now mention this switch in the revised Results text.

For our prior study (Mallik and Frank, 2022), MCI depletion in motor neurons and pan-neuronally had similar effects at the NMJ (e.g., no diminishment of EPSP). They have similar effects here too (e.g., enhancement of active zone material).

***Images aren't quite as sharp in fig 8 - use a higher res image?***

It was a fine balance for Figure 8 to clearly depict the close apposition of pre- and postsynaptic components at confocal resolution. We believe that the images for the revision Fig. 8 do this fairly well. To help, we have arrows on the images to highlight mis-appositons.

***The paper is very well referenced, but still there are two papers I would suggest that would be helpful: Joe Bateman's previous study showing MCI knockdown and and the resulting pre-synaptic effect on the nervous system in flies: doi: 10.1371/journal.pgen.1010793and more for the discussion, a recent Stephan Sigrist paper (DOI: 10.1016/j.redox.2024.103454) showing a similar accumulation of active zone material with age (i.e. why we study ROS?) in the adult brain.***

Thank you – we have added these references to the Discussion. These papers and some references suggested by Reviewer #3 have helped to contextualize our findings better, and they highlight some of the conflicting (or tissue-specific) results that other groups have documented.

***************************

***Reviewer #3: This is an interesting paper authored by Mallik and Frank. Here, they describe how knocking down ND-20L, a CI subunit, increases mitochondrial superoxide, affecting synapse function and the Drosophila neuromuscular junction (NMJ). The study targets both the presynaptic (motor neurons) and the postsynaptic (muscle) compartments, yielding contrasting effects.
When ND-20L is targeted in motor neurons, ROS levels increase, triggering a homeostatic compensatory mechanism to maintain presynaptic excitation. However, when ND-20L is targeted in muscle cells, ROS levels also rise, but muscle cells are disrupted, leading to synapse degeneration and alterations in neurotransmission.***

***The authors utilised a diverse repertoire of Drosophila reagents, including RNAi and the overexpression of various genes, to manipulate CI and superoxide levels, mitochondrial fission and fusion, and other relevant processes. Additionally, they employed several pharmacological interventions using ROS scavengers such as N-Acetyl L-cysteine amide (NACA) and inhibitors like rotenone, as well as drugs affecting calcium signalling and glycolysis. A broad range of technical approaches was used, including live imaging to detect ROS using MitoSOX, electrophysiological recordings, immunocytochemistry, and confocal microscopy to visualise and quantify presynaptic active zone proteins, postsynaptic scaffolding proteins, glutamate receptors, and mitochondrial morphology.***

***The manuscript is well written, with detailed materials and methods and well-presented data. However, I have several concerns that must be addressed, as the current version presents serious issues regarding the validity of its conclusions. I want to be clear: if the following key issues are addressed with experimental data—either using previously published/unpublished data or new experiments—I will accept the conclusions of the paper without hesitation.***

We thank Reviewer #3 for this overall positive assessment. We also want to thank Reviewer #3 for highlighting a potential issue with *ND-20L* reagent validity. This was tremendously helpful, and it led to us addressing the problem with clarity. We owe the reviewer a debt of gratitude for their careful analysis.

***Major Comments***

***1. According to FlyBase, ND-20L is a testis-specific subunit, with no or minimal expression in all other adult and larval tissues, except the imaginal disc in L3. Therefore, it is difficult to justify how CI could be depleted in motor neurons or muscle cells, or how the observed phenotypes could result from CI depletion. The short-hairpin sequence of the RNAi line used matches only ND-20L, as expected, and not ND-20, indicating that it should not deplete the non-specific testis form, ND-20.***

This is possibly the most helpful reviewer comment that we have ever received. We appreciate the opportunity to address this puzzle. In the revision, we demonstrate that the other NDUFS7 homolog, *ND-20*, is **also** depleted by the same *UAS-ND-20L[RNAi]* line. The RNAi line likely gives an **on-target** depletion for *ND-20* because of an identical stretch of 19 nucleic acids in the hairpin used to target *ND-20L*.

Reviewer #3 was correct about the conundrum of *ND-20L*, its sparse expression, and how that did not seem to fit with our dataset. There is FlyAtlas2 data on FlyBase. We also regret that we also overlooked a recent study Curcio et al., 2024, *Biophys Acta Mol Cell Res* 1871(1): 119615. Both of those sources state that *ND-20L* is mostly confined to the adult male testis. We cite both now.

The resolution boils down to one detail. The *UAS-ND-20L[RNAi]* line likely yielded robust results against MCI (via NDUFS7) because the 21/21 hairpin targeting *ND-20L*:

CTGCAGCTGCAGAAGAAGGTA (near 3’ end of *ND-20L* coding region)

is also a 19/19 hairpin against *ND-20*.

TGCAGCTGCAGAAGAAGGT (near 3’ end of *ND-20* coding region)

We report in the revision manuscript that *ND-20* message levels are down in the relevant tissues (Fig. S1). This leaves our story unchanged because the targets of the *UAS-ND-20L[RNAi]* line are both NDUFS7 homologs. As such, we have renamed this line *UAS-NDUFS7[RNAi]* in the manuscript.

***Given this, the authors must provide evidence of ND-20L expression at significant levels in the studied tissues (nervous system, muscle) and demonstrate CI depletion at the protein level. This can be done by measuring mitochondrial oxygen consumption, complex I activity, or performing Blue Native Gel Electrophoresis (BNE). I would prefer the first two methods, as they are quantitative, but I would accept BNE.***

***If obtaining sufficient material from larval muscle or nervous tissue is not feasible, an alternative—albeit less ideal—approach would be to measure CI activity in larval homogenates or adult flies using a ubiquitous driver (see PMID: 38304969, PMID: 27076081, PMID: 34977670). These experiments are essential to validate the model and determine the extent of CI depletion achieved by targeting ND-20L.***

***In my opinion, depletion should be negligible, which would invalidate the model, but I acknowledge that I may be mistaken. Thus, these experiments would be highly informative. If such data have been previously generated by the same laboratory (I could not find them in the previous Frontiers publication), they should be clearly referenced. I want to emphasise that the results must be unambiguous, as the consensus is that testis-specific CI subunits are only expressed in the testis. If this is not the case, a new consensus must be reached.***

Reviewer #3 is correct. We reasoned that the most rigorous and unbiased approach would be to recruit independent collaborators to check our reagent. We (BM and CAF) teamed up with Dr. Sajad A. Bhat and Dr. Xinnan Wang of Stanford University because of their expertise in analyzing mitochondrial function. After conducting critical experiments outlined by Reviewer #3, SAB and XW helped us to revise the manuscript, and they are now co-authors.

The data checking the validity of the *NDUFS7[RNAi]* reagent are housed in (new) Figure S1. This includes quantitative RT-PCR (qRT-PCR; Fig. S1A-D). For the qRT-PCR experiments, SAB and XW performed the experiments in triplicate or quadruplicate – starting with either larval brains (neurons) or larval fillet preps (muscle). Because of the low transcript levels of *ND-20L*, the *ND-20L* results were variable, especially in neurons. However, the *ND-20* transcript levels were unambiguously down by >50% in all tissues.

The data also report oxygen consumption rates. To measure oxygen consumption, SAB and XW conducted a Seahorse assay. They used adult flies. Low tissue volume from larvae was an experimental issue, as anticipated by Reviewer #3. They limited their analysis to the adult thorax after muscle knockdown. This is because neuronal knockdown using the *NDUFS7[RNAi]* reagent did not produce viable adults.

SAB and XW used the muscle driver (*BG57-Gal4*) and *UAS-NDUFS7[RNAi]* and collected mitochondria from adult thorax muscles. The Seahorse assay showed starkly reduced oxygen consumption rates, normalized to protein concentration and compared to driver controls (Fig. S1E-F).

***Alternatively, all experiments could be repeated using ND-30. I don't understand why the authors didn't use ND-30 instead of ND-20L. However, similar to the previous point, I would like to see evidence that this RNAi line effectively depletes CI in larvae. These kinds of experiments are important for validating the model.***

We appreciate that it was puzzling to consider our synapse data from the first submission while also knowing about the *ND-20L* expression data from elsewhere. Retrospectively, it may have been advisable for us to pursue *ND-30* as an MCI target instead of *ND-20L*. But given that the *ND-20L[RNAi]* reagent targets both *NDUFS7* homologs in *Drosophila*, that explains the results. It also explains why we observed specific mitochondrial phenotypes all along.

***2. The efficiency of the other RNAi lines and the overexpression levels of genes such as Sod2 must be verified. This can be assessed using a ubiquitous driver and larval homogenates. While not a perfect method, it would at least provide proof that the RNAi lines function as intended. I have not seen such verification in the present manuscript. Again, references to previous studies—preferably from the same laboratory but always using the same RNAi lines—would be acceptable.***

***These experiments, particularly (1), are critical, as the results presented in the manuscript contradict what is currently known about ND-20L. Therefore, it must be demonstrated that the model functions as proposed.***

There are many of them, but all the other UAS-RNAi or overexpression lines have been used and verified in previous studies. We cite primary studies for these reagents in the revised Methods section.

***3. The authors should use mitochondrially targeted catalase and repeat key experiments. They used NACA and SOD2, which have very different mechanisms of action and are localised in different cellular compartments.***

***SOD2 detoxifies superoxide in the mitochondrial matrix. NACA provides reductive power to the antioxidant system and can detoxify radicals directly, but it is unclear whether it accumulates at sufficiently high concentrations in the mitochondrial matrix to be effective.***

***Since other antioxidants, such as SOD1 or catalase, do not work, and SOD2 does—reducing superoxide but increasing H₂O₂—this suggests that superoxide produced in the matrix is responsible for the observed phenotypes. However, if that is the case, how does NACA rescue the phenotype?***

Before testing any of the ROS scavengers, we were agnostic about what may or may not reverse the phenotypes. We were not even sure that ROS was directly relevant to the synaptic phenotypes.

All available data from our study point to the mitochondrial matrix as the likely source of the radicals. This is not just because of the effectiveness of SOD2. NACA is the amide form of N-acetylcysteine. As such, it is permeable through cellular and mitochondrial membranes and can have an effect on mitochondrial processes. We cite studies to that effect in our revision.

For the revision, we did not use the mitochondrially targeted catalase line, but we did conduct alternative/additional experiments suggested by Reviewer #2 and below for Reviewer #3, Comment 4.

***MitoSox experiments in Figure S1 show that NACA reduces matrix superoxide (the MitoSox signal), further supporting the idea that matrix superoxide is the key ROS. This is mechanistically relevant, as superoxide is charged and cannot leave the mitochondria. The mitochondrially targeted catalase (available at the Bloomington Drosophila Stock Center) experiment would confirm whether the effect is specifically due to superoxide. If mitochondrial catalase does not affect the MitoSox signal and fails to rescue the phenotype, it would confirm that superoxide is the key ROS.***

***These experiments should be performed in both motor neurons and muscle. I consider these experiments essential. However, I understand that, given the extensive work already performed, the authors may prefer to state that mitochondrial superoxide is likely the cause of the observed effects but that the results remain inconclusive. If so, they should explicitly acknowledge the study's limitations and indicate that the mitochondrial catalase experiment (or overexpressing a peroxiredoxin in the mitochondria) would clarify the specific role superoxide or hydrogen peroxide involved.***

Reviewer #3 offers important insight and analysis on how best to track down the key ROS responsible for the phenotypes we have observed. It was never our intention to definitively state which source of ROS was responsible, and we regret if any comment in the original draft overstated that. Regardless of the ROS source, the synaptic effects are robust, and this is our primary scope for the paper.

We agree with the reasoning that it is likely to be mitochondrial matrix superoxide. But without definitive proof, we are comfortable stating this in the study’s Limitations and Future Directions in a new Discussion section. We were able to conduct the *Sod2* experiment for Comment 4 below, and we can add this information to the Results and Discussion.

***4. Knocking down Sod2 would further clarify the role of superoxide. If Sod2 knockdown increases the MitoSox signal, it should produce the same effects as knocking down ND-20L. If it does not increase the MitoSox signal, it would suggest that without CI depletion, superoxide levels remain very low. Again, I consider this an excellent experiment, but I understand that the authors may have a different perspective and prefer to discuss point 3 instead.***

This was a helpful and straightforward idea to test. In response, we acquired a *UAS-Sod2[RNAi]* line, and we expressed it neuronally and in muscles. We demonstrate in a new Fig. S2 that, like *UAS-NDUFS7[RNAi]*, *UAS-Sod2[RNAi]* enhances MitoSox signal in all relevant tissues examined (larval VNC, axon, NMJ, and muscle).

***Additional Concerns***

***5. The authors should specify whether they used males, females, or a mix of both. If they did not distinguish between sexes, this must be explicitly stated in the Materials and Methods. Again, ND-20L should not be expressed in females, but I am open to data that proves otherwise. If such data have already been published, they need to be referenced.***

For these larval experiments, we used both male and female larvae. We have made a note in the Methods.

In our experience, we have found that male and female larvae yield indistinguishable physiological responses at the NMJ – unless there is a relevant change of gene dosage due to dosage compensation of a factor on chromosome X. *ND-20L* may not be expressed at notable levels in females, but *ND-20* is widely expressed for both sexes. For this reason, the *UAS-NDUFS7[RNAi]* line should be effective for both.

***6. Were the cultures synchronised? If not, how do the authors ensure that control and experimental animals are at the same developmental stage? Knocking down mitochondrial genes can cause developmental delay.***

We conducted our larval analyses on wandering third instar larvae. This practice ensured a (roughly) synchronized developmental stage – even if some experimental genotypes are developmentally delayed in real time. The wandering larval stage is short, lasting a maximum of about six hours.

***7. Using red and green in figures can make interpretation difficult for colour-blind readers. Consider modifying the colour scheme to improve accessibility.***

We regret having red and green side-by-side for several of our bar graphs. Each bar graph was generated as its own image in GraphPad Prism, and those images are embedded into the figures. Rather than swap dozens of bars or graphs out of the revision figures, we ensured that each bar within the graphs is labeled with a genotype or condition. This allows accessibility for all readers. In no cases are the colors of the bar graphs essential to understanding the results.

***Minor Comments***

***1. Page 3: A reference should be added for the total number of CI subunits (42 in this paper), as different sources report varying numbers.***

Thank you. We added a reference for Garcia et al., 2017 which reported at least 42 Drosophila orthologs of the 44 human MCI subunits. We have added the “at least” qualifier.

***2. Page 3: I believed the 14 core catalytic subunits are conserved in Drosophila. Am I mistaken?***

Thank you – 14 is correct, and the Garcia reference backs it up. We fixed this for our revision.

***3. Page 3: The literature on CI depletion and ROS shows conflicting results (PMID: 36794623, PMID: 3535522, PMID: 24243023). These discrepancies should be discussed.***

We have added paragraphs about mitochondrial ROS in the Discussion, and we have focused on those *Drosophila* findings, in conjunction with Reviewer #2’s suggestions. Taking these three PMID citations together:

PMID 36794623 shows that reverse electron transfer at MCI increases ROS production and that this is concomitant with aging. Inhibition of RET decreases ROS and increases lifespan, which would suggest that ROS is “harmful” in this scenario.

The Reviewer intended to type PMID 35355221 instead of PMID 3535522. That paper suggests the opposite finding, that under stress-inducing conditions, inhibition of RET decreases ROS production and decreases lifespan – while activation of RET enhances ROS and extends lifespan. That paper would suggest that ROS is “protective.”

PMID 24243023 shows that ND-75 disruption (MCI) elevates ROS levels in muscle. That is consistent on a molecular level. But it also shows that this ROS triggers UPR activation in muscle to preserve function. That suggests that ROS is “protective” in this scenario.

***4. Figure 3, panels E-E': The images do not appear representative of a rescue phenotype. Do the authors have a better example?***

Thank you, we replaced this with a better example that demonstrates the Futsch loops.

***5. The authors mention three different temperatures, (18, 25 and 29) in Materials and Methods, but these are not referenced again. Were these different temperatures used to control gene expression?***

Apologies – we frequently use all three temperatures in our studies. But in this case, we only housed our flies at 25ºC. We have fixed the error.
